# Sulfonamides a Promising Hit for Cancer Therapy Through VEGFR-2 Inhibition

**DOI:** 10.3390/biomedicines13040772

**Published:** 2025-03-21

**Authors:** Eleftherios Charissopoulos, Eleni Pontiki

**Affiliations:** Department of Pharmaceutical Chemistry, School of Pharmacy, Faculty of Health Sciences, Aristotle University of Thessaloniki, 54124 Thessaloniki, Greece; echariss@pharm.auth.gr

**Keywords:** sulfonamide, anti-cancer, multitarget, VEGFR-2, carbonic anhydrase

## Abstract

Vascular endothelial growth factor receptor-2 (VEGFR-2), a tyrosine kinase receptor (TKR), plays a crucial role in angiogenesis and is overexpressed in most cancers. It is important for tumor angiogenesis, facilitating essential angiogenic cellular processes, such as promoting endothelial cell survival, proliferation, migration, and vascular permeability. Consequently, VEGFR-2 has become one of the main targets for anti-angiogenic therapy, with its inhibition serving as a crucial strategy for developing new drugs to mitigate angiogenesis-dependent cancers. Small-molecule drugs targeting VEGFR-2, approved by the USFDA, are exhibiting the development of drug resistance during chemotherapy, with cardiac-related side effects being consistently reported. In conclusion, it is important to develop novel strategies to enhance the efficacy of VEGFR-2 inhibitors and eliminate their adverse effects. Multifunctional drugs that target multiple pathways present a promising strategy, enhancing efficacy while minimizing side effects. Sulfonamide derivatives are extensively used in medicinal chemistry and modern drug discovery due to their variety of pharmacological activities. The present review focuses on novel compounds endowed with potential VEGFR-2 inhibition, four of which additionally present carbonic anhydrase inhibitory activity.

## 1. Introduction

### 1.1. Main Role of VEGFR-2 in Cancer: Involvement in Tumor Proliferation and Migration Across Cancer Types

Cancer is ranked as the second leading cause of mortality, according to WHO reports. The number of cancer-related deaths by 2030 will reach thirteen million worldwide [1]. Angiogenesis plays a crucial role in tumor growth and metastasis. It provides oxygen and nutrients to the tumor, enabling it to grow, survive, and spread to different parts of the body [2]. The vascular endothelial growth factor receptor-2 (VEGFR-2), as an important tyrosine transmembrane protein, is one of the main target proteins in the field of cancer treatment. VEGFs and their receptors (VEGFRs) have a crucial function in important physiological processes like angiogenesis, control of the early embryonic development of blood vessels from precursor cells, and the later formation of blood vessels from preexisting vessels. They also enhance the chemotaxis and vascular permeability of vascular endothelial cells [3,4]. The expression of VEGFR2 is located on both blood vascular and growing lymphatic vessels [5]. VEGFR-2 was found to be present in the following cancers: lung cancer, breast cancer, glioblastoma, gastrointestinal cancer, hepatocellular carcinoma, renal cell carcinoma, bladder carcinoma, and osteosarcoma [6]. In particular, there is an over-expression of VEGFR-2 in breast cancer (64.5%) and non-small-cell lung cancer (NSCLC, 54.2%) in comparison with the normal endothelial cells [7].

### 1.2. Overview of the VPF/VEGF Family and Their Key Functions

**Figure 1:**
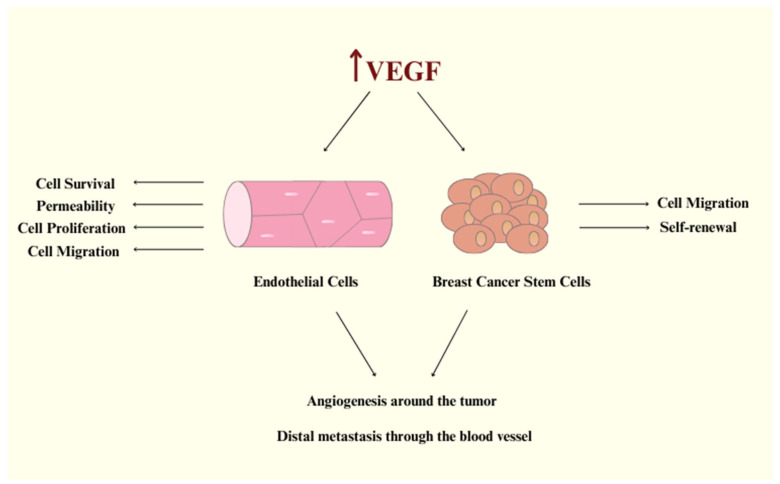
VEGF signaling promotes endothelial cell activation and tumor angiogenesis, fueling breast cancer cell migration, stemness, and metastatic spread.

Vascular endothelial growth factor-A (VEGF-A) is considered to be the founding member of the VPF/VEGF family and is responsible for increasing vascular permeability, modulating endothelial cell sprouting, mitogenesis, cell migration, and vasodilation (Figure 1) [5,8]. VEGF-A is a large, anti-parallel homodimeric peptide classified within the “Cys-loop” protein superfamily, characterized by a central cysteine knot motif where cysteine residues create intramolecular disulfide bonds upon folding [9]. This family also includes VEGF-B, VEGF-C, VEGF-D, and placenta growth factor (PIGF). VEGFs exert their biological processes by binding to VEGF receptors (VEGFRs) on target cells and triggering downstream signaling pathways. Based on their receptor binding patterns, the VEGF family can be categorized into three groups: (i) VEGF-A, which interacts with VEGFR-1 and VEGFR-2; (ii) PIGF and VEGF-B, which exclusively bind to VEGFR-1; and (iii) VEGF-C and VEGF-D, which bind to both VEGFR-2 and VEGFR-3 [5,8,10]. There have been contradictory findings between studies concerning VEGF-B. Some research found that VEGF-B has angiogenic activity, while other studies reported no angiogenic activity at all [11]. Recent advances in VEGF-B biology have revealed its key roles: it acts as a potent neuroprotective factor, displays ischemia-specific angiogenic activity in the myocardium with minimal effects on other organs, and regulates energy metabolism by controlling fatty acid uptake, as identified by Dr. Eriksson’s group [12]. Vascular endothelial growth factor-C (VEGF-C) participates in the regulation of tumor angiogenesis and lymphangiogenesis and is considered to be a multifaceted factor. VEGF-C is expressed not only in endothelial cells but also in tumor cells, where its signaling plays a crucial role in the progression of various cancer types [13]. A secreted glycoprotein called vascular endothelial growth factor-D (VEGF-D) can activate endothelium’s VEGF receptors. It operates as a mitogen for endothelial cells and promotes the development and remodeling of lymphatic and blood vessels [14]. According to some research, PlGF promotes pathological angiogenesis by triggering crosstalk between VEGFR-1 and VEGFR-2; however, other investigations did not support these conclusions [15]. Furthermore, pathological overexpression of PlGF has been reported in various tissues, including the thyroid gland, heart, lungs, skeletal muscle, and adipose tissue [16]. There are three subtypes of the VEGFRs family: VEGFR-1, VEGFR-2, and VEGFR-3 [1]. VEGFR-1 and VEGFR-2 are expressed in the normal vascular endothelium in vivo and in cultured endothelial cells. Although the majority of the functional activity results from VEGF-A binding to VEGFR-2 on the tumor endothelium, VEGF binds to VEGFR-1 with a greater affinity. Compared to VEGFR-1, VEGFR-2 is expressed in higher copy numbers [5,8]. Furthermore, VEGFR-2 controls embryonic vasculogenesis as well as tumor angiogenesis [17]. As a result, VEGFR-2 has become the primary target for anti-angiogenic therapy, with its inhibition serving as a crucial strategy for developing new drugs to combat angiogenesis-dependent cancers [1].

### 1.3. Structural Features and Activation Mechanism of VEGFR-2

**Figure 2:**
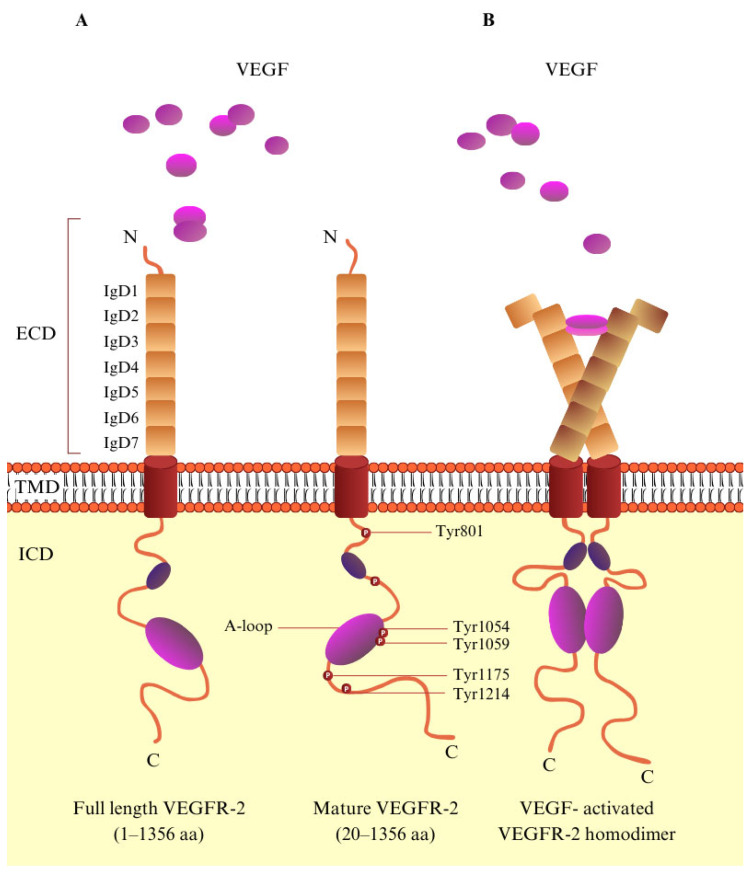
The molecular structure of VEGF/VEGFR-2. (**A**) Schematic representation of VEGFR-2. VEGFR-2 consists of a signal peptide, an extracellular domain (ECD) containing seven immunoglobulin-like subdomains (IgD1–7), a transmembrane domain (TMD), and an intracellular domain. (**B**) VEGF-activated VEGFR-2 homodimer. Upon VEGF binding to VEGFR-2, key tyrosine residues within the tyrosine kinase domain become phosphorylated, playing a vital role in regulating downstream signaling pathways.

VEGFR-2 is a tyrosine kinase receptor with a molecular mass of 230 kD and is encoded by 1356 amino acids [18]. VEGFR-2, like the majority of RTKs, is composed of three domains: the extracellular (EC), a single-pass *α*-helical transmembrane (TM), and the intracellular (IC), which includes a kinase domain, split by a 70-amino-acid insert and sequences that are required for downstream signaling [19,20,21]. The extracellular domain is one of the largest of the RTK family; it consists of seven immunoglobulin (Ig)-like domains and is highly *N*-glycosylated [19,21,22]. Through the binding of VEGF to the extracellular portion of the receptor, VEGFR-2 dimerizes. This induces the autophosphorylation of tyrosine, leading to the activation of the signaling pathway. In the structure of VEGFR-2, Tyr951, Tyr1054, Tyr 1059, Tyr1175, Tyr1214, Tyr1305, Tyr1309, and Tyr1319 are phosphorylation sites, and Tyr1054 and Tyr1059 in particular are the main sites for kinase activity (Figure 2) [23,24,25]. The conception that VEGF-A binds to a predimerized VEGFR-2 is supported by evidence showing that VEGF-A has a significantly higher affinity for VEGFR-2 dimers. Its affinity is approximately 100 times greater than that for receptor monomers [26]. Autophosphorylation of VEGFR-2 is a crucial part of the signal pathway, and therefore, efforts are being made to discover and synthesize small molecules that inhibit this reaction [23]. Furthermore, VEGFR’s-2 extracellular domain contributes significantly to VEGFR-2 angiogenetic signaling by forming lateral heterodimers with other cell surface molecules [22].

VEGFR-2 is a tyrosine kinase protein, and so it has the characteristic bilobed structure [27]. The intracellular domain, also known as the catalytic domain, contains the N-lobe (small) and C-lobe (large) [24]. Between these two lobes, in the kinase domain, there is an ATP binding cleft. An activation loop (A-loop) in the C-terminal lobe is identified by a conserved triad aspartate–phenylalanine–glycine (DFG) motif at the start of the loop. There is an active and inactive conformation of the protein kinase structure [28]. The A-loop is positioned away from the catalytic center (open conformation) in the active state (DFG-in), revealing the residues that bind the protein substrate. At the same time, it directs the catalytic aspartic acid into the ATP binding pocket. Conversely, in the inactive conformation, the A-loop adopts a closed conformation, creating a hydrophobic back pocket adjacent to the ATP binding cleft. This pocket plays a crucial role in the binding of certain tyrosine kinase inhibitors [28]. According to the binding pose, there are three types of tyrosine kinase inhibitors: type I, II, and II. At the active form ‘DFG-in’, type I inhibitors, such as sunitinib (Table 1), bind at the pocket accommodated by the adenine ring of ATP, and they form H-bonds at the binding pocket of the receptor [24,29,30]. Type II inhibitors, e.g., sorafenib, regorafenib, and tivozanib (Table 1), interact with the adjacent hydrophobic site in the ‘DFG-out’ conformation [24,30]. Finally, at the ATP binding site, type III inhibitors, such as vatalanib (Table 1), can form covalent interactions with cysteine amino acid residue [1].

### 1.4. Key Elements Involved in VEGFR-2 Inhibition

**Figure 3:**
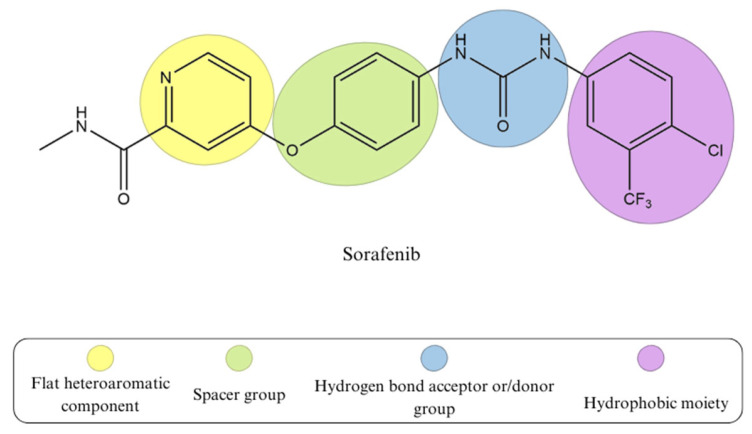
The common pharmacophoric features of sorafenib; type II VEGFR-2 tyrosine kinase inhibitor.

According to literature reports, type II kinase inhibitors are generally more significant than type I. The reason for this is that type II kinase inhibitors have slower off-rates and more particular kinase selectivity [36]. When designing VEGFR-2 inhibitors, it is essential to determine which critical structural elements interact with the highly conserved active site of the kinase domain. According to several studies, there are four main elements that contribute to inhibition (Figure 3). Firstly, a flat heteroaromatic component is essential for interacting with the hinge region. Additionally, monocyclic or bicyclic rings, known as spacer groups, occupy the gatekeeper region. Third is the existence of hydrogen bond acceptor or/donor groups that bind to the DFG motif, and lastly a terminal hydrophobic moiety to create hydrophobic interactions with the ATP binding site’s allosteric hydrophobic pocket [37,38].

### 1.5. Structural Significance of Sulfonamide Moiety and Its Role in Various Diseases

**Figure 4:**
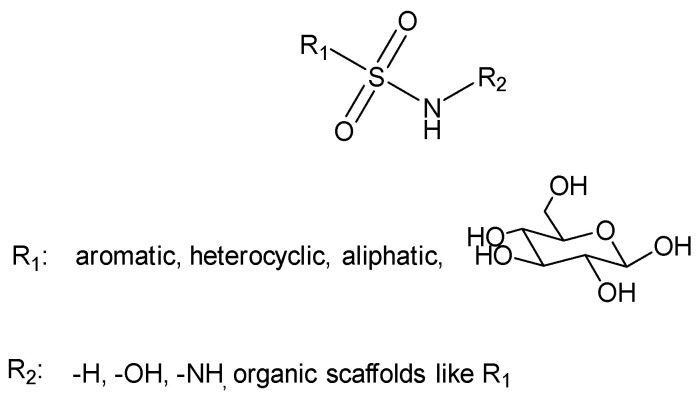
General structure of sulfonamides.

The general formula for sulfonamides and their structurally related derivatives, including sulfamates and sulfamides, is R_1_–SO_2_NHR_2_ (Figure 4). In these compounds, the functional group is either directly attached to an aromatic, heterocyclic, aliphatic, or sugar scaffold (R_1_) or is added to such a scaffold by a heteroatom, usually nitrogen or oxygen (resulting in sulfamates and sulfamides, respectively). The R_2_ group may be hydrogen or a variety of moieties that include heteroatoms (OH, NH_2_, etc.) and organic scaffolds of the types previously mentioned for R_1_ [39]. Sulfonamide derivatives are extensively used in medicinal chemistry and modern drug discovery due to their variety of pharmacological activities, such as anti-tumor, anti-bacterial, anti-HIV, and anti-inflammatory [40]. Sulfonamide moiety is an effective bioisosteric group of the carboxylic group and forms the same network of hydrogen bonds [41].

## 2. New Sulfonamide-Based VEGFR-2 Derivatives

### 2.1. Sulfonamide Derivatives with Unsubstituted Amine Group

#### 2.1.1. Sulfonamide-Linked Schiff Bases

**Figure 5:**
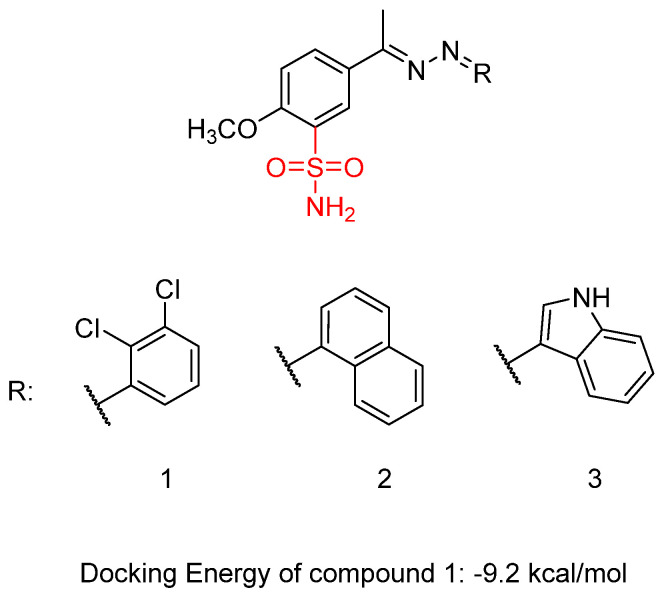
Chemical structures of sulfonamide-linked Schiff bases (**1**–**3**) [42].

Shaldam, M.M. et al. [42] synthesized and biologically evaluated a novel class of Schiff bases tethered with sulfonamide moiety. Human tumor cell lines, HepG2, human liver cancer cell line, and MCF-7, human breast cancer cell line, were used employing MTT assay to test their cytotoxic activity. Compound **1** showed the highest anti-proliferative activity against MCF-7 with IC_50_ = 0.09 μM, followed by compound **2** with IC_50_ = 0.26 μM. Compound **3** also presented promising activity, with IC_50_ = 1.11 μM. Staurosporine (IC_50_ = 3.10 μM) was used as a reference. The compounds exhibited a high anti-proliferative activity against HepG2 cell lines, with an IC_50_ range from 0.15 to 1.55 μM, compound **1** and **2** (Figure 5) having the most potent inhibition (IC_50_ = 0.15 μM) compared to staurosporine (IC_50_ = 10.42 μM). The compounds’ in vitro carbonic anhydrase (CA) and VEGFR-2 inhibitory activity was investigated, and none of them was able to inhibit the CA isoforms (*K*_I_ > 100 µM). Acetazolamide was used as control. Compound **1** demonstrated the most potent VEGFR-2 inhibitory effect, with an IC_50_ value of 23.1 ± 0.75 nM, followed by compound **2**, which exhibited an IC_50_ value of 31.1 ± 0.75 nM. Both compounds were compared to sorafenib, which showed an IC_50_ value of 29.7 ± 0.17 nM. Compound **3** (Figure 5) exhibited the lowest activity with IC_50_ = 40.1 ± 0.90 nM. Compound **1** binds to the active site of VEGFR-2 with a docking energy of −9.2 kcal/mol. Cells treated with compounds **1** and **2** showed an increased proportion in the pre-G1 phase (44.01% and 36.22%, respectively), compared to 1.87% in untreated HepG2 cells. The evaluation of drug likeness for compounds **1** and **2** was conducted using SwissADME (www.swissadme.ch, accessed on 13 March 2025). The analysis highlights a strong alignment between the majority of the predicted physicochemical properties of compounds **1** and **2** and the reported reference ranges. Compounds **1** and **2** demonstrated promising pharmacokinetic properties, including high predicted gastrointestinal absorption, no penetration of the blood–brain barrier, and no potential binding to P-glycoprotein. Furthermore, compounds **1** and **2** showed possible inhibitory effect on two P450 cytochromes (1A2 and 2C19). The reported cytotoxic activity findings against MCF7 and HepG2 cell lines provided additional insights. Based on the obtained results, the 2,3-dichloro-phenyl substitution (compound **1**) seems to favor activity (IC_50_ = 0.09 and 0.15 μΜ) compared to naphtyl- (compound **2**) or indole- (compound **3**) substitution, which led to decreased activity, respectively (compound **2** > compound **3**) (Table 2) [42].

#### 2.1.2. Isatin-Based Sulfonamides

**Figure 6:**
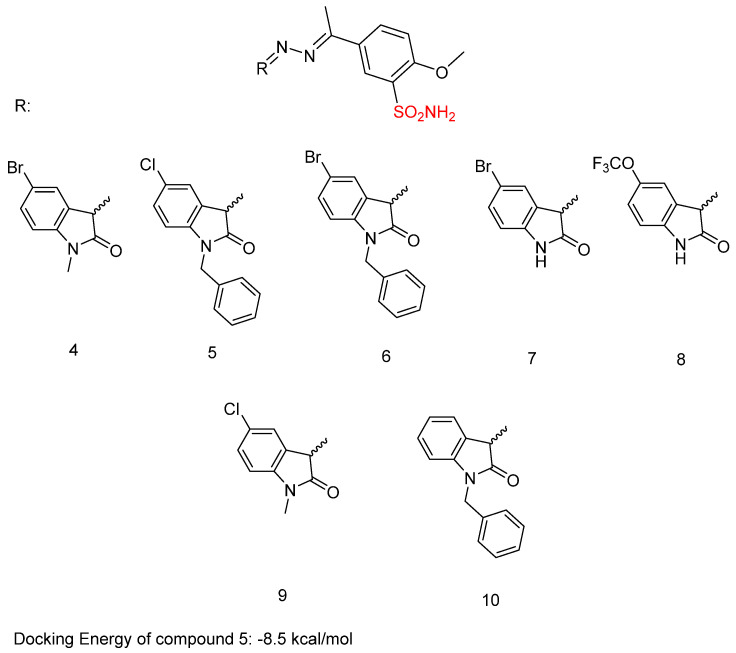
Chemical structures of isatin-based sulfonamide derivatives (**4**–**10**) [43].

In previous research, Shaldam, M.M. et al. [43] developed and analyzed a new group of isatin-based sulfonamides. The compounds were evaluated for their in vitro anti-tumor activity against the NCI panel, which includes 58 different human tumor cell lines representing nine types of cancer, at a single concentration of 10 µM. Aside from CNS cancer with compound **4** and leukemia with compounds **5** and **6**, all the compounds demonstrated the highest mean growth inhibition against breast cancer cell lines. T47D cells are the most sensitive in the breast cancer subpanel (according to GI% in vitro single-dose cellular anti-proliferative assay), showing the highest GI% response to compounds **4**, **5**, **6**, **7**, **8**, **9**, and **10** (Figure 6) with GI% values of 52, 28, 39, 54, 32, 55, and 57, respectively. To further investigate the potential anti-proliferative effects of the compounds, a dose-response analysis was conducted on T47D, breast cancer cells, using the sulforhodamine B colorimetric assay. Compounds **4**, **5**, **6**, **7**, **8**, **9**, and **10** exhibited high to moderate inhibitory activity, with IC_50_ = 10.40 ± 0.47, 3.59 ± 0.16, 16.52 ± 0.74, 5.45 ± 0.24, 24.13 ± 1.08, 1.83 ± 0.08, and 11.58 ± 0.52 μM, respectively. Compound **9** was the most potent, with IC_50_ = 1.83 ± 0.08 μM, compared with doxorubicin, with IC_50_ = 2.26 ± 0.10 μM. From the single-dose assay, the researchers noted that the 5-Br analogue was preferred over the *N*-(un)alkylated derivatives, and compound **7** (IC_50_ = 5.45 ± 0.24 µM) emerged as the most potent within the series. Among the *N*-alkylated/arylated isatin analogs with methyl or benzyl groups, the presence of a chloro substitution at position 5 resulted in the highest potency, with the *N*-methyl compound **9** showing an IC_50_ of 1.83 ± 0.08 µM and the *N*-benzyl compound **5** an IC_50_ of 3.59 ± 0.16 µM. Compounds **4**, **5**, **7**, and **9** were tested for their in vitro VEGFR-2 inhibition activities, via stopped flow assay, with IC_50_ = 30.10 ± 0.31, 23.10 ± 0.41, 56.70 ± 0.72, and 63.40 ± 0.72 nM, respectively, compound **5** having the highest inhibition (IC_50_ = 23.10 ± 0.41 nM), followed by compound **4** (IC_50_ = 30.10 ± 0.31 nM). Sorafenib was used as a standard with IC_50_ = 29.70 ± 0.17. In contrast, none of the evaluated isatin derivatives were able to inhibit the CA isoforms (*K*_I_ > 100 µM). This lack of activity may be due to steric hindrance caused by the adjacent methoxy group. T47D cells that were treated with compound **9** showed an increase in the sub-G1 and G0/G1 phases (45.88% and 68.42%, respectively), in comparison to 61.39% and 2.41%, respectively, in the control (DMSO). Compound **5** demonstrated an increase in the proportion of cells in the S and sub-G1 phases of the cell cycle, rising from 28.55% and 2.41% in untreated T47D cells to 41.05% and 39.15%, respectively. Compound **5** with the phenyl moiety attached to ethylindolin-2-one exhibited the most potent VEGFR-2 inhibition (IC_50_ = 23.10 ± 0.41 nM), while 5-chloro substitution of 1-methylindolin-2-one (compound **9**) reduced VEGFR-2 inhibitory activity (IC_50_ = 63.40 ± 0.72 nM) compared to the 5-Br substitution (compound **4**) (IC_50_ = 30.10 ± 0.31 nM) (Table 3). Docking studies of compound **5** in the active site of the VEGFR-2 receptor resulted in a docking score of −8.5 kcal/mol [43].

#### 2.1.3. Quinazoline Sulfonamide Derivatives

**Figure 7:**
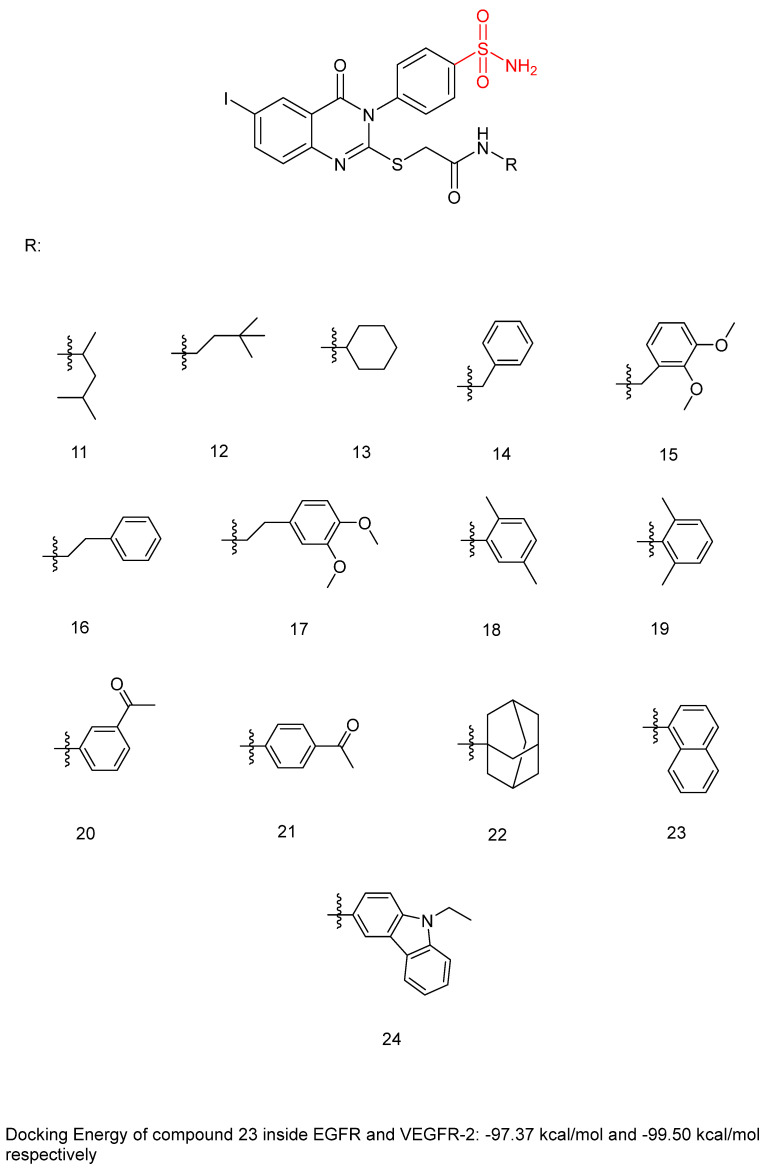
Chemical structures of quinazoline sulfonamide derivatives **11** to **24** [44].

Ghorab, M.M. et al. [44] developed and analyzed a new group of quinazoline sulfonamide conjugates. Four different human tumor cell lines—HepG2, MCF-7, HCT-116, human colon cancer cell line, and A549, adenocarcinomic human alveolar basal epithelial cells—were used via MTT assay to test their cytotoxicity. The results demonstrated that the majority of the compounds had growth-inhibitory activity ranging from good to poor against the studied cancer cell types. Compounds **12**, **13**, **14**, **15**, **16**, **18**, **19**, **20**, **22**, **23**, and **24** (Figure 7) exhibited the strongest anti-cancer activity against HepG2, with IC_50_ = 0.1163 ± 0.02, 0.1943 ± 0.02, 0.1707 ± 0.02, 0.2232 ± 0.02, 0.4273 ± 0.03, 0.4484 ± 0.02, 0.4660 ± 0.02, 0.3434 ± 0.02, 0.3076 ± 0.02, 0.3425 ± 0.02, and 0.1952 ± 0.02 µM, respectively, comparing with sorafenib (IC_50_ = 0.400 ± 0.03) and erlotinib (IC_50_ =0.773 ± 0.07). Compounds **12**, **14**, **17**, **18**, **20**, **22**, **23**, and **24** displayed the highest anti-cancer activity against the MCF-7 cell line, with IC_50_ = 0.4092 ± 0.02, 0.4620 ± 0.02, 0.1781 ± 0.02, 0.1773 ± 0.02, 0.4588 ± 0.02, 0.4029 ± 0.05, 0.0977 ± 0.01, and 0.3618 ± 0.02 µM, respectively, comparing with sorafenib (IC_50_ = 0.404 ± 0.03 µM) and erlotinib (IC_50_ = 0.549 ± 0.05 µM). Compound **13** was the most potent, achieving an IC_50_ = 0.0977 µM. In the evaluation against HCT-116, compounds **11**, **12**, **13**, **14**, **17**, **18**, **19**, **20**, **21**, **23**, and **24** (Figure 7) exhibited outstanding anti-cancer activity, with IC_50_ = 0.2771 ± 0.02, 0.1985 ± 0.02, 0.2865 ± 0.02, 0.3792 ± 0.02, 0.1451 ± 0.01, 0.3391 ± 0.05, 0.2106 ± 0.02, 0.1704 ± 0.02, 0.2216 ± 0.02, 0.2000 ± 0.02, and 0.3202 ± 0.02 µM, respectively, using sorafenib (IC_50_ = 0.558 ± 0.05 µM) and erlotinib (IC_50_ = 0.820 ± 0.06 µM) as references. Against A549, compounds **14**, **16**, and **20** demonstrated the most potent anti-cancer effects, with IC_50_ values of 0.4511, 0.1145, and 0.4100 µM, respectively, comparing with sorafenib (IC_50_ = 0.505 ± 0.05 µM) and erlotinib (IC_50_ = 0.1391 ± 0.01 µM). Compounds **13**–**16**, **20**, **22**, and **23** showed excellent cytotoxic activity and were tested for their inhibitory activities against mutant EGFR^T790M^ kinase inhibitory activities, using erlotinib as a reference with IC_50_ = 0.2420 µM. Compound **23** showed the best inhibitory effect of EGFR^T790M^. Compounds **14**, **20**, and **22** effectively inhibited EGFR^T790M^, with IC_50_ values of 0.3898, 0.3683, and 0.3516 µM, respectively. Compounds **12**, **14**, **15**, **17**, and **22**–**24** were selected for evaluation of their VEGFR-2 inhibitory potential using the AlphaScreen system (PerkinElmer, Waltham, MA, USA) with an anti-phosphotyrosine antibody. Sorafenib was used as standard with IC_50_ = 0.1400 ± 0.01 µM. All the compounds had an IC_50_ range of 0.0523–0.393 µM, with compounds **17** (IC_50_ = 0.0984 µM) and **23** (IC_50_ = 0.0523 µM) exhibiting stronger EGFR^T790M^ inhibitory activity than sorafenib. In conclusion, compound **23** demonstrated outstanding dual inhibitory activity against EGFR^T790M^ and VEGFR-2 with IC_50_ = 0.0728 and 0.0523 µM, respectively, and cytotoxic activity against MCF-7 with IC_50_ = 0.0977 µM. Compound **23** binds inside EGFR with a docking energy of −97.37 kcal/mol and inside VEGFR-2 with a docking energy of −99.50 kcal/mol. MCF-7 cells were treated with compound **23** to evaluate its impact on the cell cycle. The results indicate that compound **23** arrests the cell cycle’s G2/M phase. Compound **23** was evaluated for its cytotoxicity on normal cell line Hek-293 to assess its safety. The results showed low cytotoxicity on the normal Hek-293 cell line (IC_50_ = 0.6144 µM). Consequently, it is 1.79, 6.29, 3.07, and 1.20 times more toxic against HepG2, MCF-7, HCT-116, and A549 cells, respectively, compared to Hek-293 cells. A benzyl group (compound **14**) (IC_50_ = 0.3898 ± 0.02 μM against EGFR^T790M^) may play a crucial role in enhancing cytotoxic activity and EGFR^T790M^ inhibition, whereas lengthening the alkyl chain with an extra carbon (compound **16**) (IC_50_ = 1.2567 ± 0.15 μM against EGFR^T790M^) appears to reduce these effects. Methyl substitutions on different positions of the phenyl ring influence cytotoxic activity. Compound **18** (IC_50_ = 0.1773 ± 0.02 μM against MCF-7), with ortho and meta methyl substitutions, demonstrated slightly better cytotoxicity in the MCF-7 cell line compared to compound **19** (IC_50_ = 1.6433 ± 0.25 μΜ against MCF-7), which bears two ortho methyl groups (Table 4). The presence of ortho and meta methoxy groups in compound **15** appears to slightly reduce both VEGFR-2 inhibitory activity and cytotoxicity, compared to compound **14**, which is unsubstituted. Compound **23**, with a naphthalene substitution, demonstrates the most potent cytotoxic profile and exhibits the strongest dual inhibition of VEGFR-2 and EGFR^T790M^, according to the published results [44].

#### 2.1.4. 1,5-Diaryl-1,2,4-Triazole-Tethered Sulfonamide Derivatives

**Figure 8:**
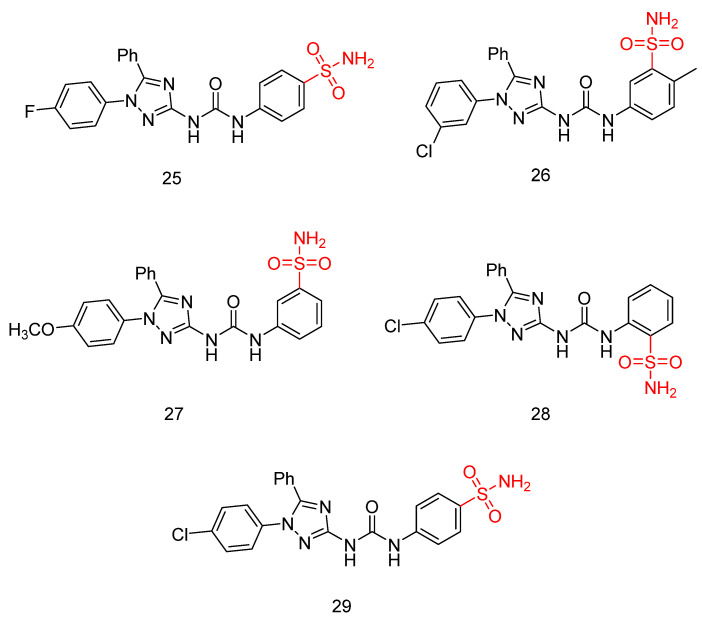
Chemical structures of 1,5-diaryl-1,2,4-triazole-tethered sulfonamide derivatives (**25**–**29**) [45].

In their recent research, Elsawi, A.E. et al. [45] synthesized a new set of 1,5-diaryl-1,2,4-triazole-tethered sulfonamide derivatives and tested their potential dual CA IX/XII and VEGFR-2 inhibitory activities. All the synthesized compounds were tested for their selective inhibition for the two transmembrane cancer-associated *h*CA IX and XII over the cytosolic physiologically prominent *h*CA I and II isoforms (applying stopped-flow CO_2_ hydrase assay) and demonstrated moderate to excellent results. To further investigate their in vitro anti-proliferative activity, they submitted the compounds to the NCI Developmental Therapeutic Program for screening of growth-inhibitory potential against diverse cancer cell panels. The anti-proliferative assays were performed following the protocol established by the Drug Evaluation Branch of the NCI in Bethesda. In particular, 60 cancer cell lines were used to screen the entire set of compounds at 10 μM. The most potent compounds were used to investigate their potential VEGFR-2 inhibition utilizing a colorimetric enzyme-linked immunosorbent assay (ELISA). Compound **25** exhibited the highest inhibitory activity with IC_50_ = 26.3 ± 0.4 nM compared to sunitinib (IC_50_ = 39.7 ± 2 nM), followed by compound **26** with an IC_50_ value of 96.2 ± 2 nM. Compounds **27**, **28**, and **29** exhibited modest inhibitory effects, with an IC_50_ = 183 ± 8.1, 271 ± 14.0, and 318 ± 13 nM, respectively. They have been tested in vitro for anti-proliferative action against breast MCF-7 and T47D cancer cell lines, using the MTT assay protocol. Staurosporine was used as standard for MCF-7 and T47D, with IC_50_ values of 2.17 ± 0.09 and 7.12 ± 0.31 μM, respectively. Compounds **25**, **26**, **27**, **28**, and **29** (Figure 8) exhibited excellent to good anti-proliferative activity on both MCF-7 and T47D cancer cell lines, with IC_50_ = 0.66 ± 0.04, 10.9 ± 0.62, 1.06 ± 0.04, 2.19 ± 0.09, and 2.17 ± 0.09 μM, respectively, against MCF-7 and 4.51 ± 0.2, 17.9 ± 0.78, 2.34 ± 0.09, 2.97 ± 0.12, and 2.84 ± 0.11 μM, respectively, against T47D. A cell growth inhibition assay was conducted for compounds **25**, **27**, **28**, and **29** on the non-tumorigenic human breast epithelial cell line (MCF 10A). Compounds **25**, **27**, and **29** demonstrated remarkable selectivity, with index values of 38.75, 44.81, and 29.93, respectively. Compound **28** also exhibited good selectivity. Thus, these compounds can selectively target breast cancer cells while sparing non-tumorigenic breast tissue. Compound **25** demonstrated strong inhibitory activity against hypoxia-induced *h*CA IX and VEGFR-2, with KI and IC_50_ values of 4.7 nM and 26.3 nM, respectively. It also showed impressive submicromolar anti-proliferative effects on MCF-7 cell lines, with an IC_50_ of 0.66 ± 0.04 µM. By docking the co-crystallized ligands in the active sites of the corresponding enzyme receptors, they verified the molecular docking process. Compound **25** was chosen to undergo molecular docking in the three enzymes: *h*CA isoform IX, *h*CA isoform XII, and VEGFR-2. The small RMSD values of 0.504, 0.98, and 0.20 Å observed between the docked poses and the co-crystallized ligands of *h*CA IX, XII, and VEGFR-2, respectively, indicate an almost identical alignment, confirming the reliability of the applied setup for the planned docking study. Based on the obtained results, a compound bearing a para-sulfonamide substitution (compound **29**) (IC_50_; 318 ± 13 nM), along with ortho substitution in compound **28** (IC_50_ = 271 ± 14.0 nM), resulted in a decrease in VEGFR-2 inhibitory activity. Replacing the 4-Cl of the phenyl ring of compound **29** (IC_50_ = 318 ± 13 nM) with 4-F, resulting in compound **25** (IC_50_ = 26.3 ± 0.4 nM), significantly enhanced VEGFR-2 inhibitory activity (Table 5) [45].

### 2.2. N-Substituded Sulfonamides

#### 2.2.1. Sulfonamide–Triazole–Glycoside Hybrid Derivatives

**Figure 9:**
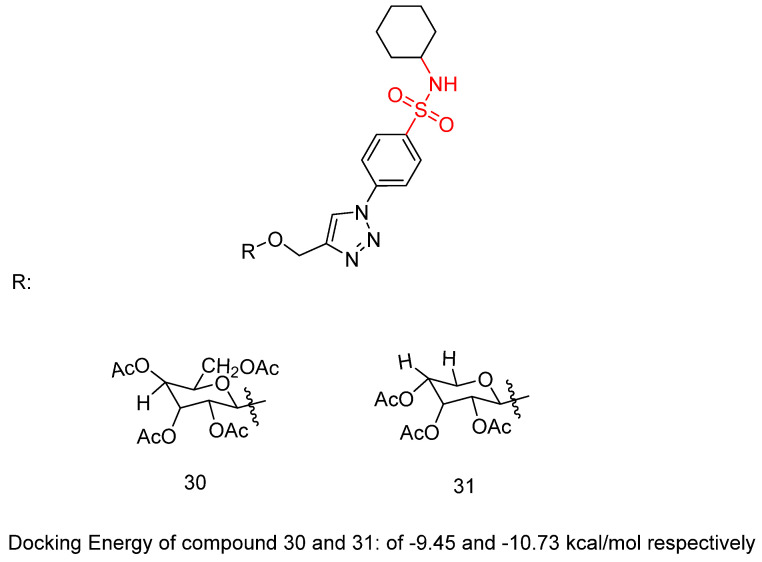
Chemical structures of sulfonamide–triazole–glycoside hybrid derivatives **30** and **31** [46].

Abbas, H.A.S. et al. [46] constructed a novel class of sulfonamide–triazole–glycoside hybrid derivatives. The synthesized compounds were specifically designed to investigate their potential to inhibit cell proliferation in four different cancer cell lines—lung (A-549), liver (HepG-2), breast (MCF-7), and colon (HCT-116)—through the MTT assay. Compounds **30** and **31** (Figure 9) exhibited promising activity against HepG-2 and MCF-7 (IC_50_ = 10.45 ± 0.13 and 8.39 ± 0.20 μM, respectively, against HepG-2 and 20.31 ± 0.66 and 21.15 ± 2.45 μM, respectively, against MCF-7), comparing with doxorubicin (IC_50_ = 13.76 ± 0.45 and 17.44 ± 0.46 μM against HepG-2 and MCF-7, respectively). Due to their remarkable cytotoxic results, compounds **30** and **31** were evaluated for their in vitro inhibitory potency against VEGFR-2 and the carbonic anhydrase isoforms *h*CA IX and *h*CA XII using sorafenib and SLC-0111 as reference compounds. Compounds **30** and **31** demonstrated promising potency, with higher selectivity of compound **31** than **30** against VEGFR-2, *h*CA IX, and *h*CA XII (IC_50_ = 1.33 μM, 66, and 7.6 nM, respectively, for 31 and 0.38 μM, 40, and 3.2 nM, respectively, for 32), comparing with sorafenib and SLC-0111 (IC_50_ = 0.43 μM, 53, and 4.8 nM, respectively). Compound **31** is able to arrest MCF-7 cells in the G2/M phase of the cell cycle. Compound **31** was tested for its impact on Bax, Bcl-2, and p53 levels in MCF-7 cells. Treatment of MCF-7 cells with compound **31** for 24 h resulted in a 6.2-fold increase in Bax levels (271.45 pg/mL) compared to untreated control cells (43.66 pg/mL). Moreover, Bcl-2 protein expression was reduced by 2.6 times in MCF-7 cells treated with compound **31**, decreasing from 8.51 to 3.27 ng/mL. Furthermore, compound **31** increased p53 protein levels by 7.4 times in treated MCF-7 cells compared to 125.40 pg/mL in control cells (Table 6). Among all the synthesized compounds, the cyclohexane substitution in the sulfonamide moiety exhibited the most favorable effects. In the glycoside group, the presence of an acetoxymethyl group at the 2-position, combined with the configuration of the acetate group at the 3-position, led to a reduction in cytotoxic activity, *h*CA IX and *h*CA XII inhibition, as well as VEGFR-2 inhibition. Compounds **30** and **31** displayed promising binding inside the VEGFR-2 active site with a docking energy of −9.45 and −10.73 kcal/mol, respectively [46].

#### 2.2.2. N-(4-(6-amino-5-cyano-4-(3-fluorophenyl)pyridin-2-yl)phenyl)-4-methylbenzenesulfonamide

**Figure 10:**
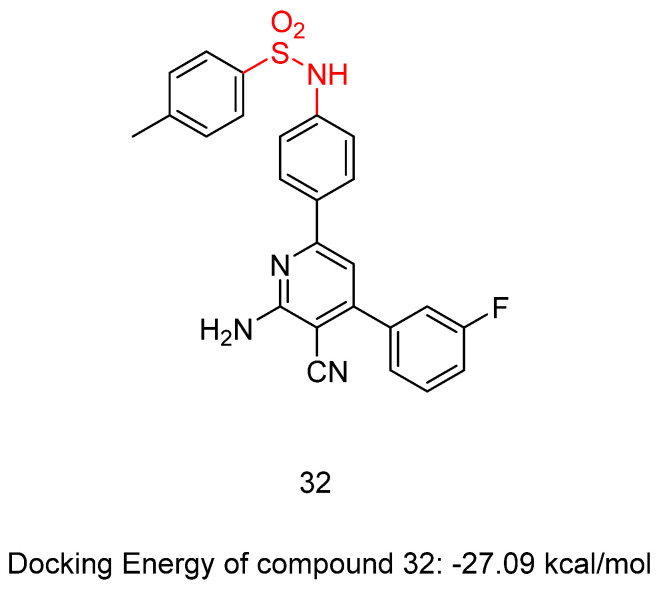
Chemical structures of N-(4-(6-amino-5-cyano-4-(3-fluorophenyl)pyridin-2-yl)phenyl)-4-methylbenzenesulfonamide, compound **32** [47].

New sulfonamide derivatives were synthesized and assessed by Ahmed, M.F. et al. [47]. All of the derivatives were tested in vitro, with a full NCI panel five-dose assay, against 60 lines of human cancer cells, and the results indicated that compound **32** (Figure 10) was the most potent. Compound **32** demonstrated strong anti-proliferative activity, with GI50 values between 1.06 and 8.92 μM against most of the cancer cell lines tested, arresting the cell cycle at the G2/M phase. The inhibitory effect of compound **32** against VEGFR-2 was assessed, with sorafenib (IC_50_ = 4.58 μM) used as a reference. The compound demonstrated significant inhibitory activity, exhibiting an IC_50_ value of 3.62 μM. Compound **32** induced apoptosis in UO-31 cells by increasing the expression of BAX, caspase-3, and P53 and suppressing the expression of Bcl-2 (Table 7). Compound **32** demonstrated a strong fitting to the VEGFR-2 active site with a docking score of −27.09 kcal/mol [47].

#### 2.2.3. N-(substituted)-4-(thiazolo [4,5-b]quinoxalin-2(3H)-ylideneamino)-benzenesulfonamide Derivatives

**Figure 11:**
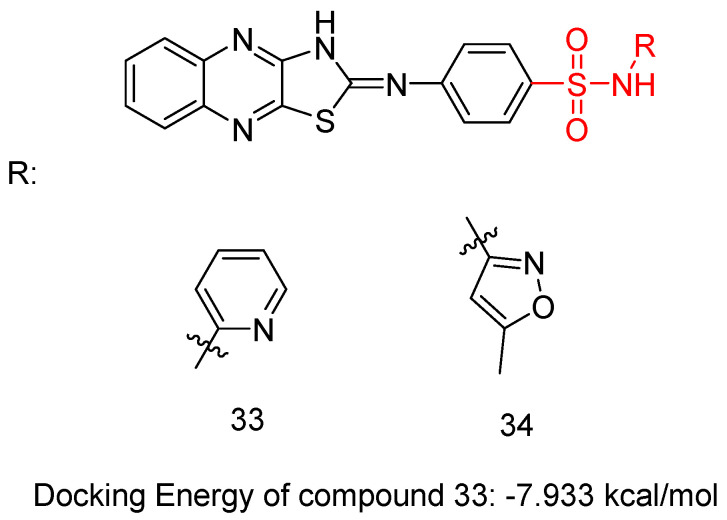
Chemical structures of N-(substituted)-4-(thiazolo [4,5-b]quinoxalin-2(3H)-ylideneamino)-benzenesulfonamide derivatives [48].

In a recent study, El-Hazek, R.M.M. et al. [48] conducted a thorough study of a novel set of *N*-*(substituted*)-4-(thiazolo [4,5-b]quinoxalin-2*(3H)-*ylideneamino)-benzenesulfonamide derivatives and tested their cytotoxic anti-cancer activity against the human HepG2 cell line. Compounds **33** and **34** (Figure 11) showed potential inhibitory effects against VEGFR-2 (61.04 and 83.35 nM, respectively), comparable to standard sorafenib (51.41 nM), using the VEGFR-2 Kinase Assay Kit. Compound **33** exhibited remarkable inhibition of the human HepG2 cell line (IC_50_ = 4.31 μM), comparing with sorafenib (IC_50_ = 2.97 μM) (Table 8). A comparative evaluation of cardiomyocyte viability between compound **33** and sorafenib was conducted using H9C2 cell cultures, and the results revealed that compound **33** exhibited 2.93 times lower cytotoxicity than sorafenib. The researchers carried out an in vivo study to establish the myocardium safety of compound **33** on irradiated mice (8Gy). Results indicated normal cardiac enzyme function (CK) and serum catalase activity, with considerable decreases in LDH, cardiac TNF-α, and caspase-9 levels, along with its effectiveness in inhibiting the expression of hepatic VEGF. Thus, compound **33** exhibited a greater in vitro myocardium cytoprotective effect compared to the commonly used anti-HCC drug, sorafenib. The primary benefit of using compound **33** in this study is its low radiation-induced cardiotoxic potential, as it reduced the elevated levels of pro-apoptotic, pro-inflammatory, and oxidative mediators in the myocardium of irradiated mice. In compound **33**, pyridine substitution significantly enhanced cytotoxic activity and increased VEGFR-2 inhibition compared to the 3-methylisoxazole substitution in compound **34**. Compound **33** mainly occupies the catalytic site of the VEGFR-2 receptor, with a docking energy of −7.933 kcal/mol [48].

#### 2.2.4. Sulfamoyl-Substituted Hydrazone and Pyrazolidinedione Derivatives

**Figure 12:**
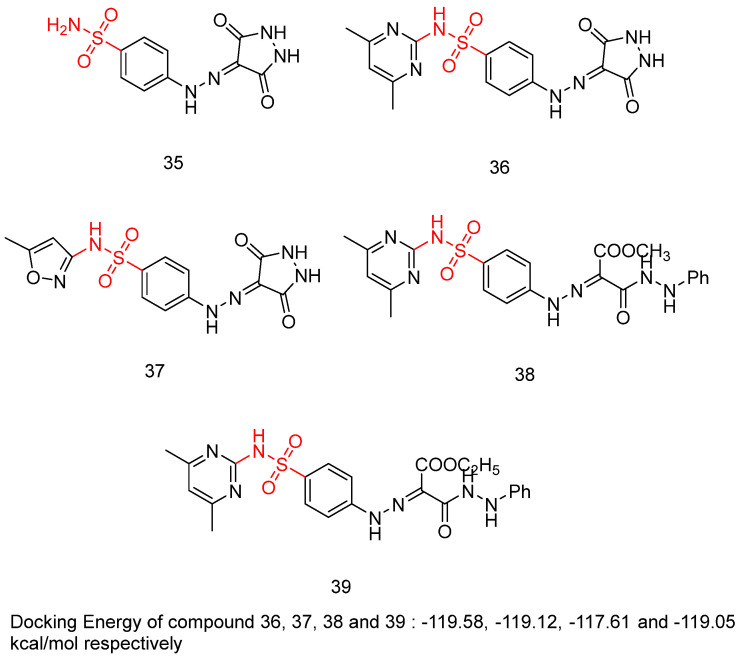
Chemical structures of sulfamoyl-substituted hydrazone and pyrazolidinedione derivatives [49].

Sayed, A.M. et al. [49] produced a novel class of diethyl or dimethyl 2-(2-(4-substitutedsulfamoylphenyl)-hydrazineylidene) malonate derivatives, 4-((3,5-dioxopyrazolidin-4-yl)diazenyl)benzenesulfonamide derivatives, and 3,5-dioxopyrazolidin-4-yl derivatives. Three human tumor cell lines—HepG2, HCT-116, and MCF-7—were used via MTT assay to test their cytotoxic activity. The results indicated that most of the compounds displayed excellent to modest growth-inhibitory activity. Sorafenib (IC_50_ = 9.18 ± 0.6, 5.47 ± 0.3, and 7.26 ± 0.3 in HepG2, HCT-116, and MCF-7, respectively) and doxorubicin (IC_50_ = 7.94 ± 0.6, 8.07 ± 0.8, and 6.75 ± 0.4 in HepG2, HCT-116, and MCF-7, respectively) were included in the experiments as references. Compounds **35**, **36**, **37**, **38**, and **39** (Figure 12) demonstrated the most potent inhibitory activity against the three cell lines HepG2, HCT-116, and MCF-7 with IC_50_ = 17.06 ± 1.5, 12.48 ± 1.1, 27.48 ± 2.2 μM; 6.43 ± 0.5, 9.66 ± 0.8, 10.57 ± 0.9 µM; 8.65 ± 0.7, 7.49 ± 0.6, 14.29 ± 1.3 µM; 11.17 ± 1.0, 19.52 ± 1.7, 21.65 ± 1.9 μM; and 8.97 ± 0.7, 10.13 ± 0.9, 13.82 ± 1.1 µM, respectively (Table 9). A test for the cytotoxicity of compounds **35**, **36**, **37**, **38**, and **39** against VERO cell lines was conducted and showed low toxicity, with IC_50_ = 30.09 ± 0.31, 39.42 ± 0.31, 36.33 ± 0.32, 63.12 ± 0.43, and 59.76 ± 0.43 μM, respectively, in comparison to the cytotoxicity against the cancer cell line that varies from 6.43 to 27.48 μM. Additionally, the most potent anti-proliferative derivatives, **35**, **36**, **37**, **38**, and **39**, were tested for their VEGFR-2 inhibitory activities, by using an anti-phosphotyrosine antibody with the AlphaScreen system (PerkinElmer, USA). Compounds **35**, **36**, **37**, **38**, and **39** exhibited high to good inhibitory activity, with IC_50_ = 0.23 ± 0.03, 0.14 ± 0.02, 0.15 ± 0.02, 0.17 ± 0.02, and 0.15 ± 0.02 µM, respectively, comparing with sorafenib (IC_50_ = 0.10 ± 0.02 µM). Compounds **36**, **37**, and **39** emerged as the most potent derivatives, exhibiting VEGFR-2 inhibition with IC_50_ values of 0.14 ± 0.02, 0.15 ± 0.02, and 0.15 ± 0.02 µM, respectively. The non-substituted sulfonamide derivative, compound **35**, exhibited the weakest cytotoxic activity and VEGFR-2 inhibition. Methyl substitution in the carboxyl group of compound **38** reduced cytotoxicity but did not significantly affect VEGFR-2 inhibition compared to compound **39**, which has an ethyl substitution. Docking studies for compound **36**, **37**, **38**, and **39** revealed high affinity values of −119.58, −119.12, −117.61, and −119.05 kcal/mol, respectively [49].

## 3. Conclusions

With the identification of associated genes, transcription factors, and signaling pathways, angiogenesis suppression has become a promising therapeutic strategy in the ongoing battle against cancer. VEGFR-2 and its signaling pathway via VEGF have a vital role in tumor angiogenesis, which consequently leads to the discovery of new molecules targeting this receptor in cancer therapy. This is substantiated by the large number of clinically tested and approved compounds for the treatment of angiogenesis-related diseases. In this review, we focus on the role of the sulfonamide scaffold in modulating VEGFR-2 inhibition for the development of novel and potent therapeutical agents. It was discovered that a number of significant inhibitors were effective against various cancer types. Several of the synthesized compounds were tested for their potential dual VEGFR-2 and carbonic anhydrase inhibition [42,43,45,46], with promising results demonstrated solely by Elsawi, A.E. et al. and Abbas, H.A.S. et al. [45,46], specifically for compounds **25** (IC_50_: 26.3 ± 0.4 nM against VEGFR-2 and 8.3 and 4.7 nM against *h*CA IX and *h*CA XII, respectively) and **26** (IC_50_: 96.2 ± 2 nM against VEGFR-2 and 67.2 and 61.0 nM against *h*CA IX and *h*CA XII, respectively). Also promising were compounds **30** (IC_50_: 1.33 ± 0.10 μM against VEGFR-2 and 66 and 7.6 nM against *h*CA IX and *h*CA XII, respectively) and **31** (IC_50_: 0.38 ± 0.14 μM against VEGFR-2 and 40 and 3.2 nM against *h*CA IX and *h*CA XII, respectively) demonstrated promising results. Compounds **25** and **26** are non-substituted sulfonamides, whereas compounds **30** and **31** contain a cyclohexane substitution in their molecular structure. Compounds **1**, **5**, **17**, **23**, **25**, **31**, and **32** exhibited remarkable VEGFR-2 inhibition, surpassing the reference compounds, with IC_50_ values 23.1 ± 0.75 nM, 23.10 ± 0.41 nM, 0.0984 ± 0.01 μΜ, 0.0523 ± 0.01 μΜ, 26.3 ± 0.4 nM, 0.38 ± 0.14 μΜ, and 3.62 ± 0.04 μΜ, respectively. Compounds **1**, **5**, **17**, **23**, and **25** are non-substituted sulfonamide derivatives, with the sulfonamide moiety attached to a phenyl ring. In compounds **1** and **5**, a methoxy group is positioned ortho to the sulfonamide group. Additionally, compounds **31** and **32** are substituted sulfonamides, with compound **31** containing a cyclohexane group and compound **32** featuring a 4-methylbenzene group. These compounds hold potential for future research as drug candidates. The most promising compounds from this article are presented in Table S1 (Supplementary Information). Moreover, the effectiveness between the compounds in each series is compared in the Supplementary Information using graphs.

Future studies should focus on improving the structure of sulfonamide analogs to improve their pharmacokinetic profile and their efficacy to ensure a successful clinical translation.

## Figures and Tables

**Table 1:** Important drugs approved or/and under clinical development.

Drug	Structure	Year of Approval	References
**Sorafenib**	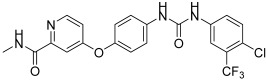	2005	Escudier, B. et al. [31]
**Sunitinib**	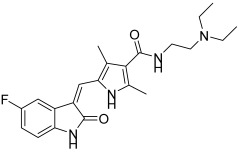	2006	Motzer, R.J. et al. [32]
**Regorafenib**	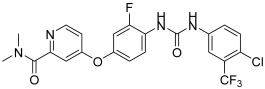	2012	Majithia, N. et al. [33]
**Tivozanib**	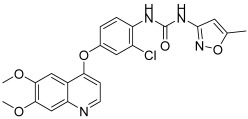	2021	Aref, M. et al. [34]
**Vatalanib**	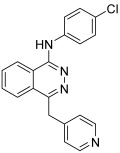	Phase III clinical trial	Scott, E.N. et al. [35]

**Table 2:** Bioassay results for compounds **1**–**3** and reference compounds [42].

Compound	MTT Assay Against Human Tumor Cell Lines HepG2 and MCF-7 (IC_50_)	CA IX and CA XII Inhibition	K_I_	VEGFR-2 Inhibition	IC_50_
**1**	0.15 μM and 0.09 μM, respectively		*K*_I_ > 100 µM		23.1 ± 0.75 nM
**2**	0.15 μM and 0.26 μM, respectively		*K*_I_ > 100 µM		31.1 ± 0.75 nM
**3**	1.55 μM and 1.11 μM, respectively		*K*_I_ > 100 µM		40.1 ± 0.90 nM
Reference: Staurosporine	10.42 μM (HepG2) 3.10 μM (MCF-7)	Reference: Acetazolamide	0.025 µM (CA IX) 0.006 µM (CA XII)	Reference: Sorafenib	29.7 ± 0.17 nM

**Table 3:** Bioassay results for most promising compounds and reference compounds [43].

Compound	Sulforhodamine B Colorimetric Assay Against T47D Breast Cancer Cells (IC_50_)	CA I, CA II, and CA IX Inhibition	K_I_	VEGFR-2 Inhibition	IC_50_
**4**	10.40 ± 0.47 μM		*K*_I_ > 100 µM		30.10 ± 0.31 nM
**5**	3.59 ± 0.16 μM		*K*_I_ > 100 µM		23.10 ± 0.41 nM
**7**	5.45 ± 0.24 μM		*K*_I_ > 100 µM		56.70 ± 0.72 nM
**9**	1.83 ± 0.08 μM		*K*_I_ > 100 µM		63.40 ± 0.72 nM
Reference: Doxorubicin	2.26 ± 0.10 μM	Reference: Acetazolamide	0.25 (CA I) 0.012 (CA II) 0.026 (CA IX)	Reference: Sorafenib	29.70 ± 0.17 nM

**Table 4:** Bioassay results for most promising compounds and reference compounds [44].

Compound	MTT Assay Against Human Tumor Cell Lines HepG2, MCF-7, HCT-116, and A549 (IC_50_)	VEGFR-2 and EGFR^T790M^ Inhibition	IC_50_
**12**	0.1163 ± 0.02, 0.4092 ± 0.02, 0.1985 ± 0.02, and 1.8986 ± 0.25 μM, respectively		0.2193 ± 0.02 μM and NT, respectively
**14**	0.1707 ± 0.02, 0.4620 ± 0.02, 0.3792 ± 0.02, and 0.4511 ± 0.05 μM, respectively		0.2510 ± 0.02 and 0.3898 ± 0.02 μM, respectively
**15**	0.2232 ± 0.02, 0.6939 ± 0.07, 0.5872 ± 0.05, and 0.7887 ± 0.07 μM, respectively		0.3931 ± 0.02 and 0.6615 ± 0.05 μM, respectively
**17**	1.1923 ± 0.10, 0.1781 ± 0.02, 0.1451 ± 0.01, and 1.7428 ± 0.15 μM, respectively		0.0984 ± 0.01 μM and NT, respectively
**22**	0.3076 ± 0.02, 0.4029 ± 0.05, 1.1217 ± 0.15, and 0.8211 ± 0.07 μM, respectively		0.2174 ± 0.02 and 0.3516 ± 0.02 μM, respectively
**23**	0.3425 ± 0.02, 0.0977 ± 0.01, 0.2000 ± 0.02, and 0.5134 ± 0.05 μM, respectively		0.0523 ± 0.01 and 0.0728 ± 0.01 μM, respectively
**24**	0.1952 ± 0.02, 0.3618 ± 0.02, 0.3202 ± 0.02, and 1.1600 ± 0.25 μM, respectively		0.1921 ± 0.02 μM and NT, respectively
Reference: Sorafenib and Erlotinib	0.400 ± 0.03 (HepG2) and 0.773 ± 0.07 μM (HepG2), respectively 0.404 ± 0.03 (MCF-7) and 0.549 ± 0.05 μM (MCF-7), respectively 0.558 ± 0.05 (HCT-116) and 0.820 ± 0.06 μM (HCT-116), respectively 0.505 ± 0.05 (A549) and 0.1391 ± 0.01 (A549), respectively	Reference: Sorafenib (VEGFR-2) and Erlotinib (EGFR^T790M^)	0.1400 ± 0.01 and 0.2420 ± 0.02 μM, respectively

**Table 5:** Bioassay results for most promising compounds and reference compounds [45].

Compound	MTT Assay Against Human Tumor Cell Lines MCF-7 and T47D (IC_50_)	*h*CA IX and *h*CA XII Inhibition	K_I_	VEGFR-2 Inhibition	IC_50_
**25**	0.66 ± 0.04 and 4.51 ± 0.2 μM, respectively		*K*_I_: 8.3 and 4.7 nM, respectively		26.3 ± 0.4 nM
**26**	10.9 ± 0.62 and 17.9 ± 0.78 μM, respectively		*K*_I_: 67.2 and 61.0 nM, respectively		96.2 ± 2 nM
Reference: Staurosporine	3.18 ± 0.18 (MCF-7) and 7.12 ± 0.31 μM (T47D)	Reference: Acetazolamide	25.0 (*h*CA IX) and 5.7 nM (*h*CA XII)	Reference: Sunitinib	39.7 ± 2 nM

**Table 6:** Bioassay results for most promising compounds (**30**–**31**) and reference compounds [46].

Compound	MTT Assay Against Human Tumor Cell Lines A-549, HepG2, MCF-7, and HCT-116 (IC_50_)	*h*CA IX and *h*CA XII Inhibition	K_I_	VEGFR-2 Inhibition	IC_50_
**30**	20.45 ± 0.28, 10.45 ± 0.13, 20.31 ± 0.66, and 32.05 ± 0.42 μM, respectively		IC_50_: 66 and 7.6 nM, respectively		1.33 ± 0.10 μM
**31**	19.81 ± 0.65, 8.39 ± 0.20, 21.15 ± 2.45, and 23.60 ± 0.22 μΜ, respectively		IC_50_: 40 and 3.2 nM, respectively		0.38 ± 0.14 μM
Reference: Doxorubicin (HepG2, MCF-7) and Sunitinib (A-549, HCT-116)	13.76 ± 0.45 (HepG2), 17.44 ± 0.46 (MCF-7), 10.14 ± 0.50 (A-549), and 9.67 ± 0.22 μM (HCT-116)	Reference: SLC-0111	53 (*h*CA IX) 4.8 nM (*h*CA XII)	Reference: Sorafenib	0.43 ± 0.10 μM

**Table 7:** Bioassay results for most promising compounds and reference compounds [47].

Compound	NCI Panel Five-Dose Assay, Against 60 Lines of Human Cancer Cells	VEGFR-2 Inhibition	IC_50_
**32**	GI50 values between 1.06 and 8.92 mM		3.62 ± 0.04 μM
		Reference: Sorafenib	4.58 ± 0.05 μM

**Table 8:** Bioassay results for most promising compounds (**33**–**34**) and reference compounds [48].

Compound	Cytotoxic Anti-Cancer Activity Against Human HepG2 Cell Line (IC_50_)	VEGFR-2 Inhibition	IC_50_
**33**	4.31 μM		61.04 ± 2.60 nM
**34**	>100 μM		83.35 ± 3.70 nM
Reference: Sorafenib	2.97 μM	Reference: Sorafenib	51.41 ± 2.30 nM

**Table 9:** Bioassay results for most promising compounds and reference compounds [49].

Compound	MTT Assay Against Human Tumor Cell Lines HepG2, HCT-116, and MCF-7 (IC_50_)	VEGFR-2 Inhibition	IC_50_
**35**	17.06 ± 1.5, 12.48 ± 1.1 and 27.48 ± 2.2 μΜ, respectively		0.23 ± 0.03 μΜ
**36**	6.43 ± 0.5, 9.66 ± 0.8 and 10.57 ± 0.9 μM, respectively		0.14 ± 0.02 μΜ
**37**	8.65 ± 0.7, 7.49 ± 0.6 and 14.29 ± 1.3 μM, respectively		0.15 ± 0.02 μΜ
**38**	11.17 ± 1.0, 19.52 ± 1.7 and 21.65 ± 1.9 μM, respectively		0.17 ± 0.02 μΜ
**39**	8.97 ± 0.7, 10.13 ± 0.9 and 13.82 ± 1.1 μΜ, respectively		0.15 ± 0.02 μΜ
Reference: Sorafenib and Doxorubicin	9.18 ± 0.6 (HepG2) and 7.94 ± 0.6 μM (HepG2), respectively 5.47 ± 0.3 (HCT-116) and 8.07 ± 0.8 μM (HCT-116), respectively 7.26 ± 0.3 (MCF-7) and 6.75 ± 0.4 μM (MCF-7), respectively	Reference: Sorafenib	0.10 ± 0.02 μΜ

## Supplementary Materials

The following supporting information can be downloaded at: https://www.mdpi.com/article/10.3390/biomedicines13040772/s1, Figure S1: IC_50_ values for compound **1**–**3** reported by Shaldam, M.M. et al. [42]; Figure S2: VEGFR-2 IC_50_ inhibition for compound **1**–**3** reported by Shaldam, M.M. et al. [42]; Figure S3: IC_50_ values for T47D inhibition by compounds **4**–**10** reported by Shaldam, M.M. et al. [43]; Figure S4: VEGFR-2 IC_50_ inhibition for compounds **4**, **5**, **7** and **9** reported by Shaldam, M.M. et al. [43]; Figure S5: IC_50_ values for HepG2, MCF-7, HCT-116 and A549 inhibition by compounds **11**–**24** reported by Ghorab, M.M et al. [44]; Figure S6: VEGFR-2 IC_50_ inhibition for compounds **12**, **14**, **15**, **17**, **22**, **23** and **24** reported by Ghorab, M.M et al. [44]; Figure S7: EGFR^T790M^ IC_50_ inhibition for compounds **13**, **14**, **15**, **16**, **20**, **22** and **23** reported by Ghorab, M.M et al. [44]; Figure S8: IC_50_ values for MCF-7 and T47D inhibition by compounds **25**–**29** reported by Elsawi, A.E et al. [45]; Figure S9: VEGFR-2 IC_50_ inhibition for compounds **25**–**29** reported by Elsawi, A.E et al. [45]; Figure S10: K_I_ values for hCA IX hCA XII inhibition by compounds **25**–**29** reported by Elsawi, A.E et al. [45]; Figure S11: IC_50_ values for A549, HepG2, MCF-7 and HCT-116 inhibition by compounds **30** and **31** reported by Abbas, H.A.S et al. [46]; Figure S12: VEGFR-2 IC_50_ inhibition for compounds **30** and **31** reported by Abbas, H.A.S et al. [46]; Figure S13: K_I_ values for hCA IX hCA XII inhibition by compounds **30** and 31 reported by Abbas, H.A.S et al. [46]; Figure S14: VEGFR-2 IC_50_ inhibition for compound **32** reported by Ahmed, M.F et al. [47]; Figure S15: IC_50_ values for HepG2 inhibition by compounds **33** and 34 reported by El-Hazek, R.M.M et al. [48]; Figure S16: VEGFR-2 IC_50_ inhibition for compounds 33 and 34 reported by El-Hazek, R.M.M et al. [48]; Figure S17: IC_50_ values for HepG2, HCT-116 and MCF-7 inhibition by compounds **35**–**39** reported by Sayed, A.M et al. [49]; Figure S18: VEGFR-2 IC_50_ inhibition for compounds **35**–**39** reported by Sayed, A.M et al. [49]; Table S1: Consolidated table of sulfonamides, bioassays performed and results on the most promising compounds and used reference compounds.

## Abbreviations

The following abbreviations are used in this manuscript:

ATP	Adenosine Triphosphate
CA	Carbonic Anhydrase
CNS	Central Nervous System
CK	Creatine Kinase
EC	Extracellular
EGFR	Epidermal Growth Factor Receptor
HepG2	Hepatoblastoma cell line, human liver cancer cell line
IC	Intracellular
Ig	Immunoglobulin
LDH	Lactate Dehydrogenase
MCF-7	Michigan Cancer Foundation-7, human breast cancer cell line
NSCLC	Non-Small-Cell Lung Cancer
PIGF	Placenta Growth Factor
RTK	Receptor Tyrosine Kinase
TM	Transmembrane
TNF-a	Tumor Necrosis Factor Alpha
VEGF	Vascular Endothelial Growth Factor
VEGF-A	Vascular Endothelial Growth Factor-A
VEGF-B	Vascular Endothelial Growth Factor-B
VEGF-C	Vascular Endothelial Growth Factor-C
VEGF-D	Vascular Endothelial Growth Factor-D
VEGFR-2	Vascular Endothelial Growth Factor Receptor-2
VPF	Vascular Permeability Factor
WHO	World Health Organization
