# Supplementary material for: Sulfonamides a Promising Hit for Cancer Therapy Through VEGFR-2 Inhibition
Source: Biomedicines. 2025 Mar 21;13(4):772. doi: 10.3390/biomedicines13040772 (PMC12025213; doi:10.3390/biomedicines13040772)
Supplement: Supplementary file 1 [file biomedicines-13-00772-s001.zip › biomedicines-3502150-supplementary.pdf]

Supplementary Information

Graphs of the biological activities of the compounds studied.

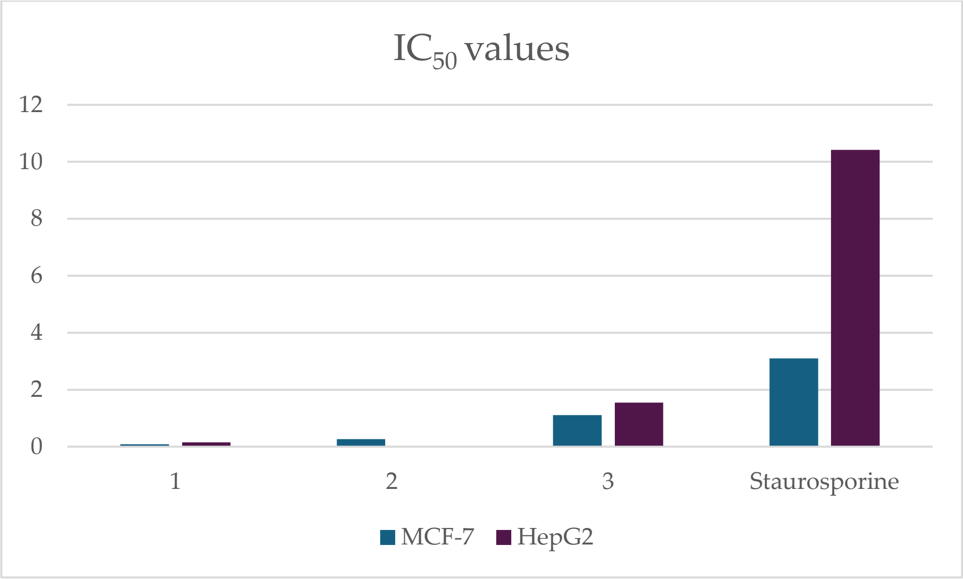

Figure S1. IC_{50} values for compound 1-3 reported by Shaldam, M.M. et al. [42]

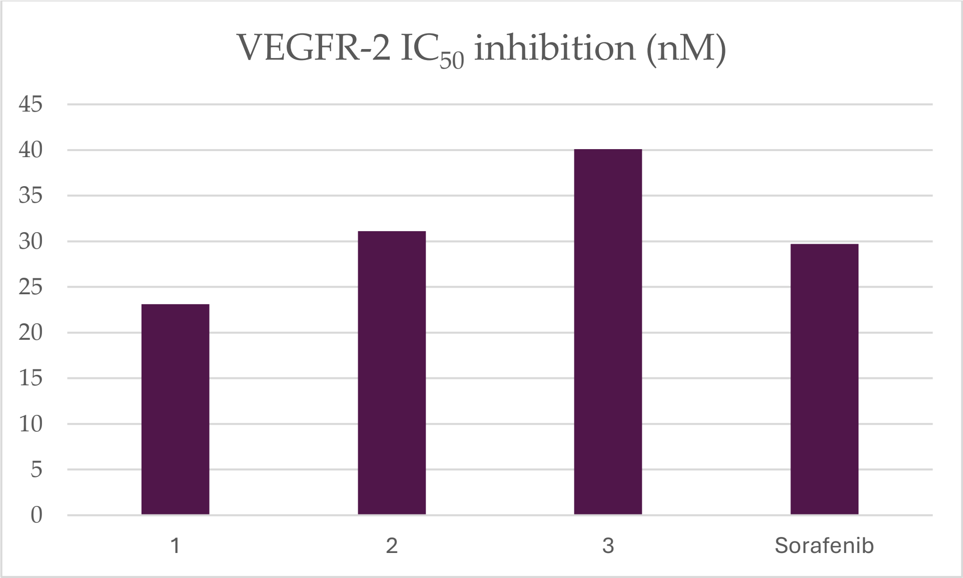

Figure S2. VEGFR-2 IC_{50} inhibition for compound 1-3 reported by Shaldam, M.M. et al. [42]

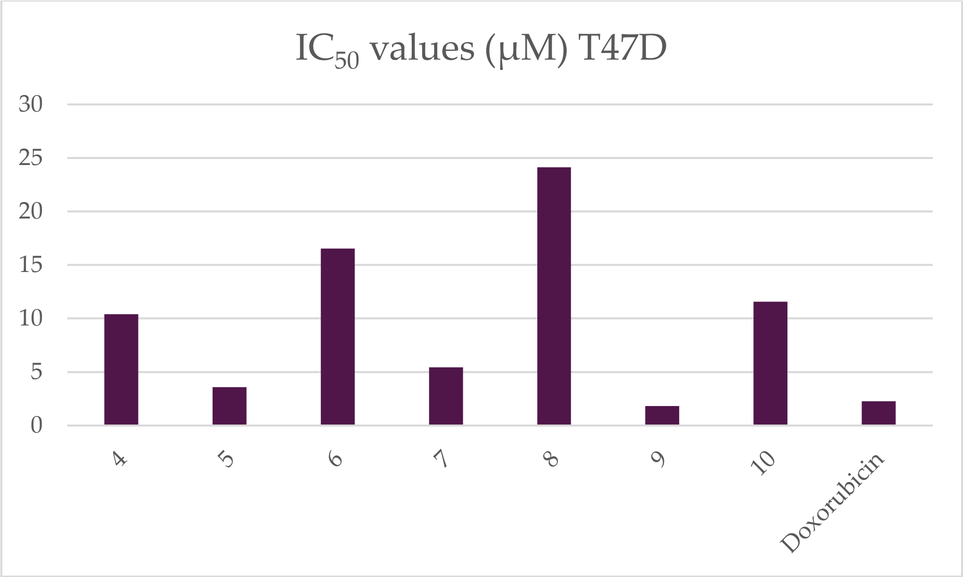

Figure S3. IC_{50} values for T47D inhibition by compounds 4-10 reported by Shaldam, M.M. et al. [43]

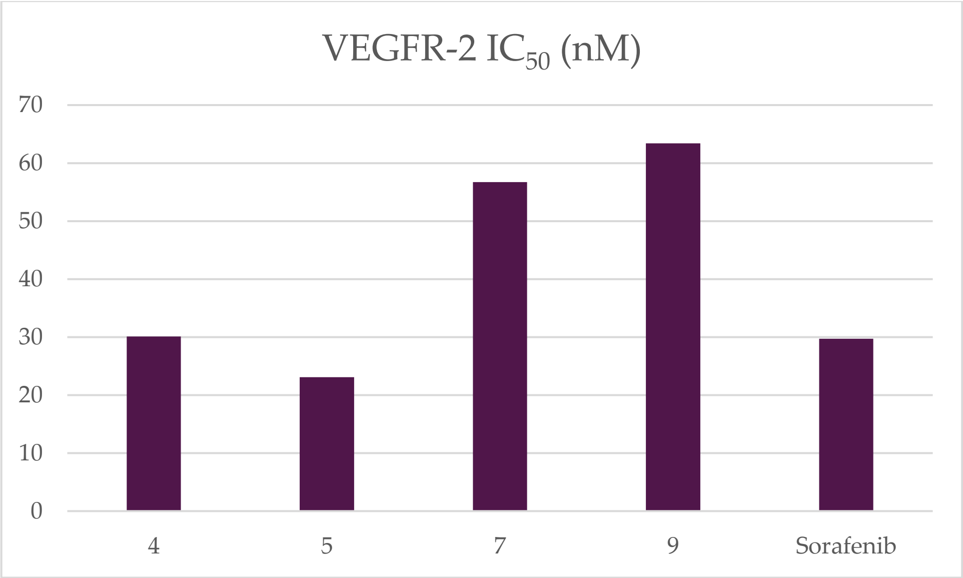

Figure S4. VEGFR-2 IC_{50} inhibition for compounds 4, 5, 7 and 9 reported by Shaldam, M.M. et al. [43]

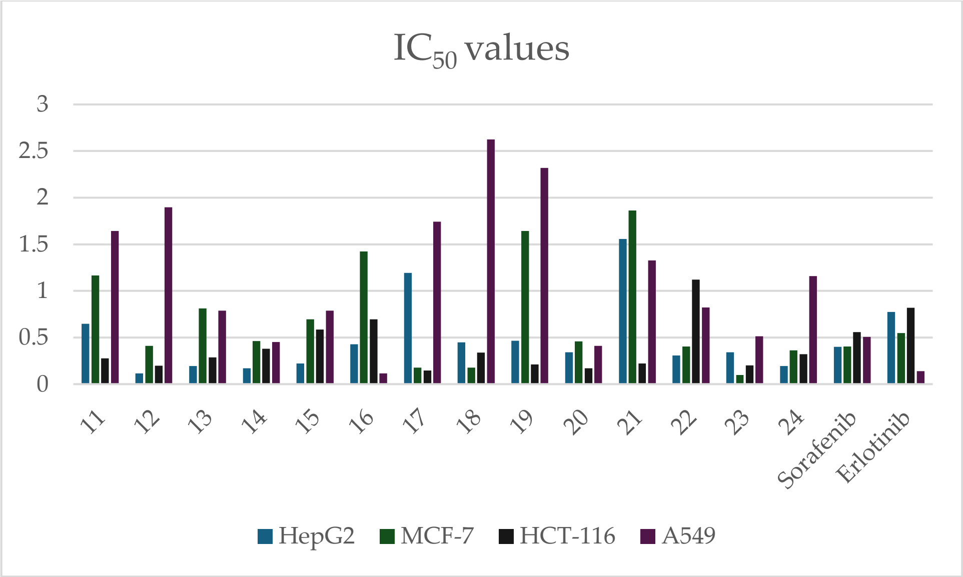

Figure S5. IC_{50} values for HepG2, MCF-7, HCT-116 and A549 inhibition by compounds 11-24 reported by Ghorab, M.M et al. [44]

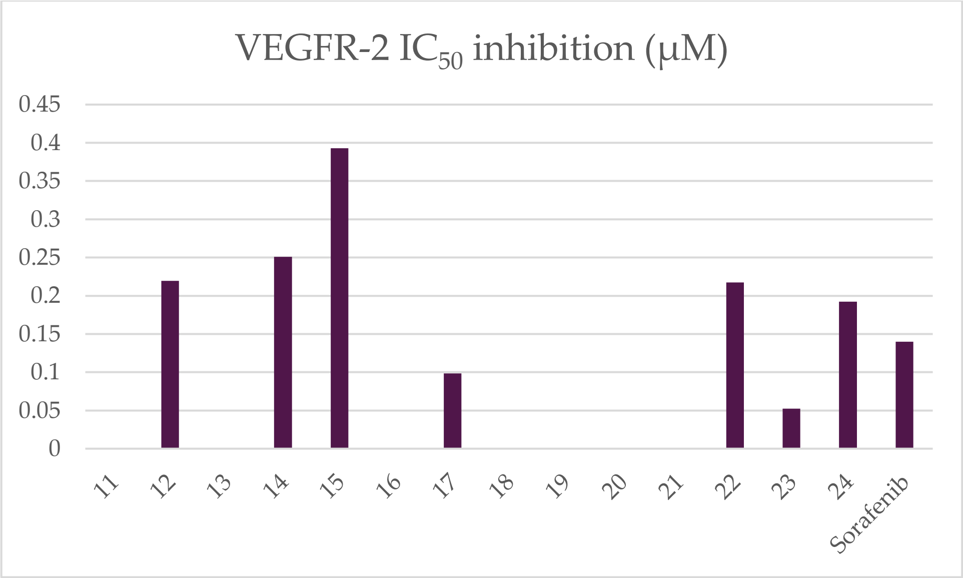

Figure S6. VEGFR-2 IC_{50} inhibition for compounds 12, 14, 15, 17, 22, 23 and 24 reported by Ghorab, M.M et al. [44]

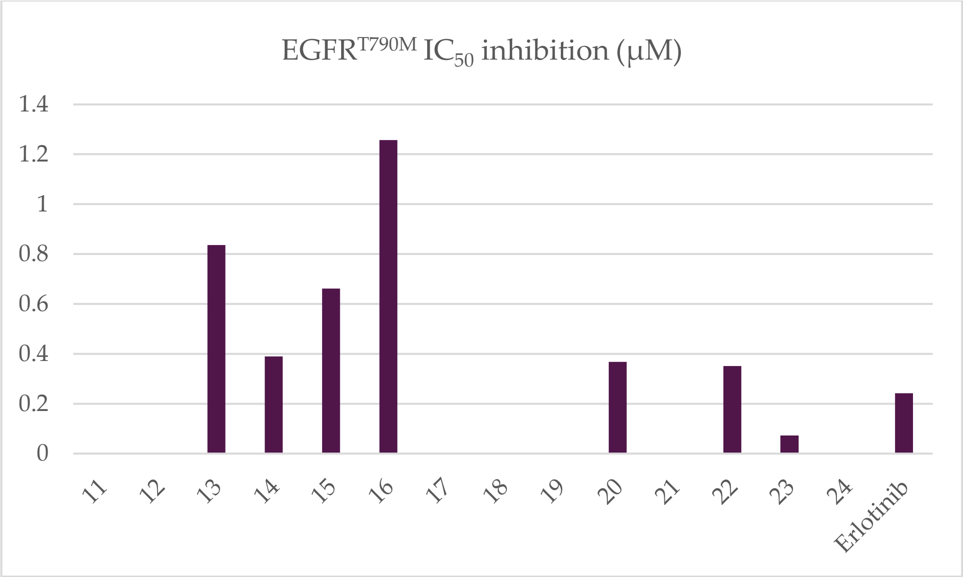

Figure S7. EGFR^{T790M} IC$_{50}$ inhibition for compounds 13, 14, 15, 16, 20, 22 and 23 reported by Ghorab, M.M et al. [44]

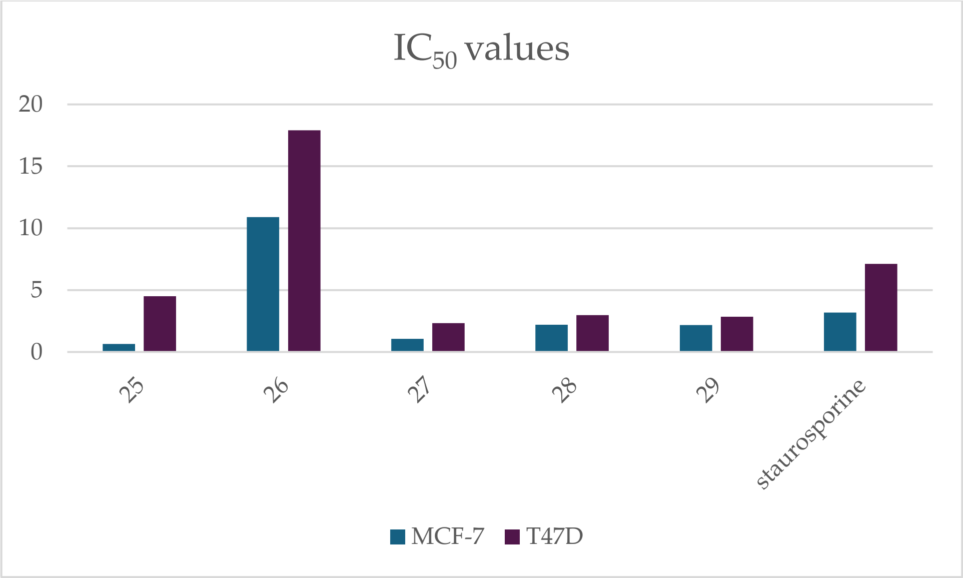

Figure S8. IC$_{50}$ values for MCF-7 and T47D inhibition by compounds 25-29 reported by Elsawi, A.E et al. [45]

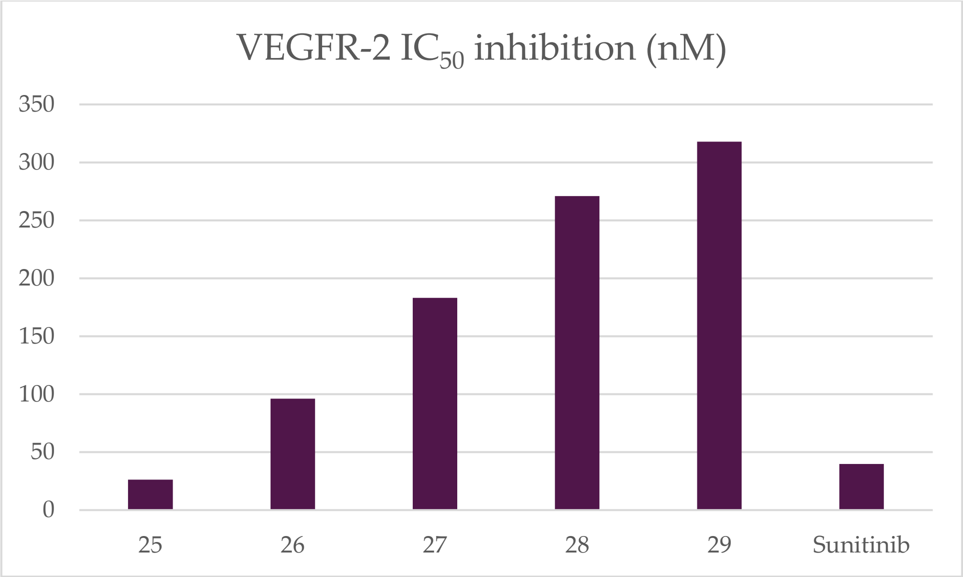

Figure S9. VEGFR-2 IC_{50} inhibition for compounds 25-29 reported by Elsawi, A.E et al. [45]

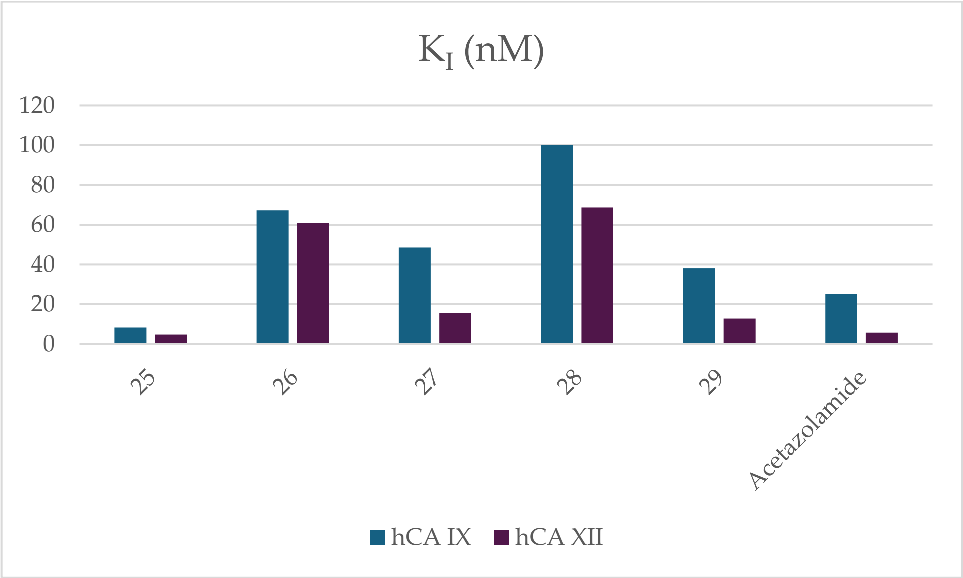

Figure S10. K_I values for hCA IX hCA XII inhibition by compounds 25-29 reported by Elsawi, A.E et al. [45]

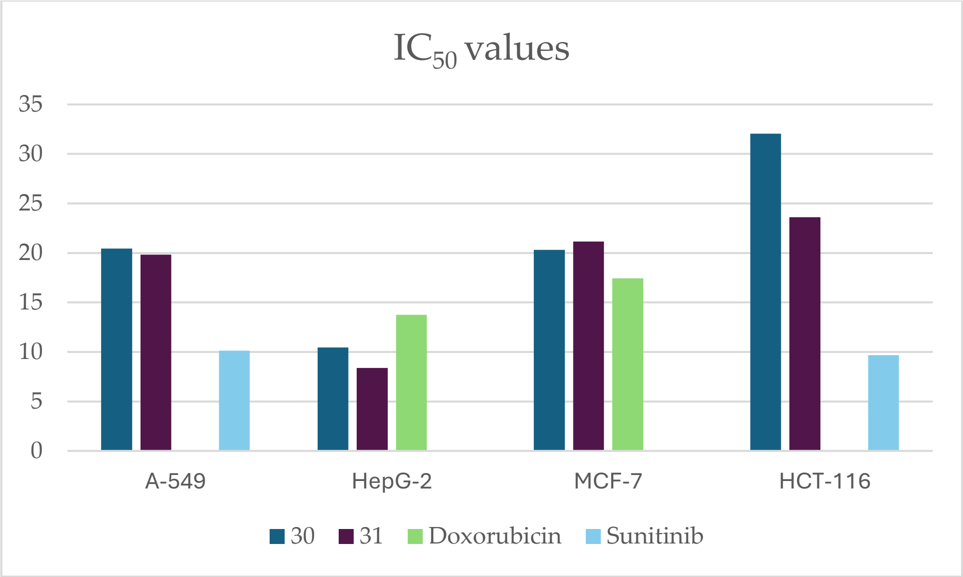

Figure S11. IC_{50} values for A549, HepG2, MCF-7 and HCT-116 inhibition by compounds 30 and 31 reported by Abbas, H.A.S et al. [46]

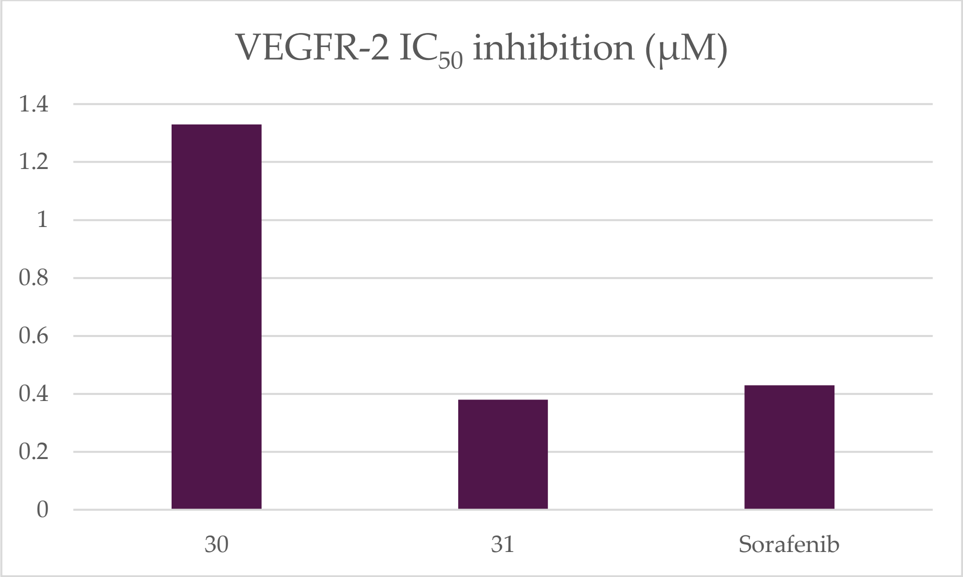

Figure S12. VEGFR-2 IC_{50} inhibition for compounds 30 and 31 reported by Abbas, H.A.S et al. [46]

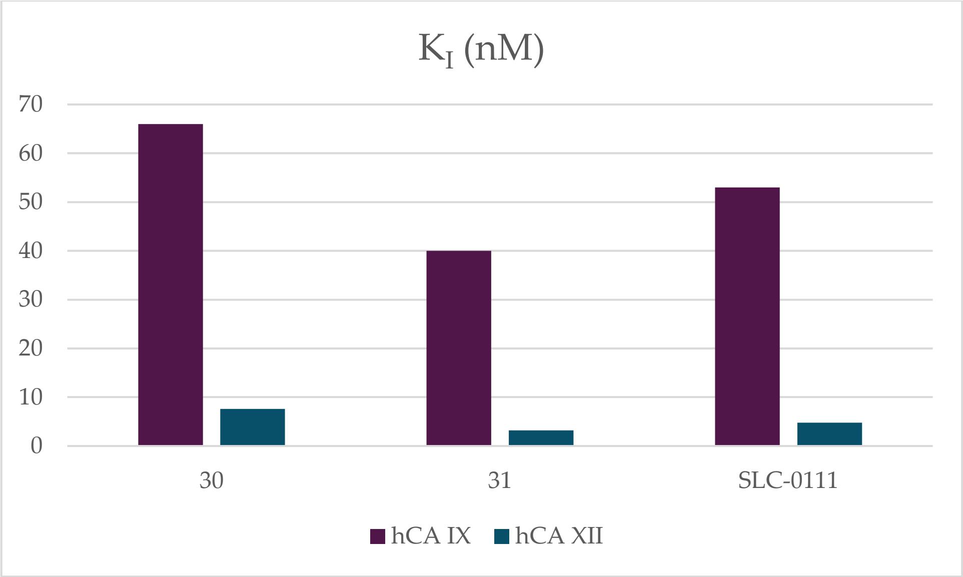

Figure S13. K_I values for hCA IX hCA XII inhibition by compounds 30 and 31 reported by Abbas, H.A.S et al. [46]

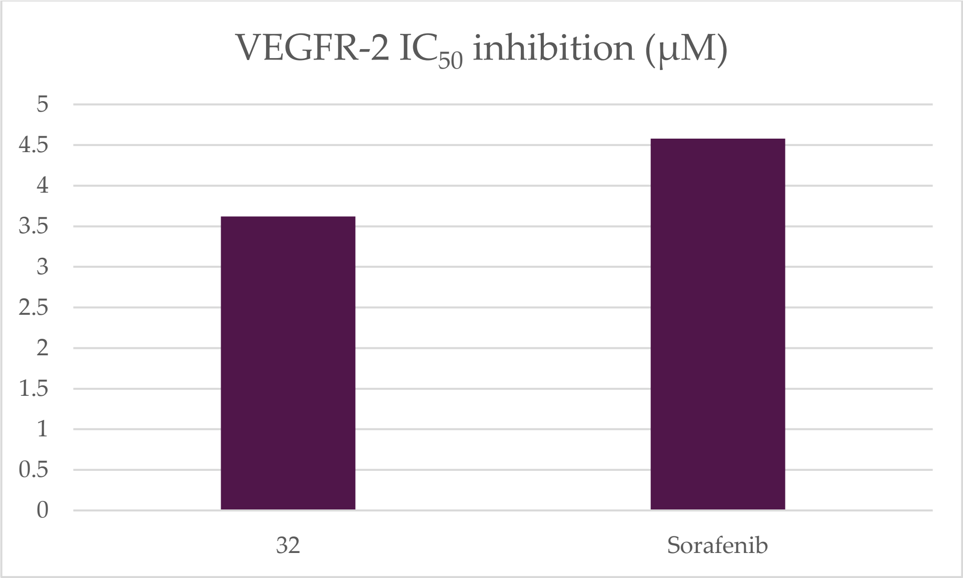

Figure S14. VEGFR-2 IC_{50} inhibition for compound 32 reported by Ahmed, M.F et al. [47]

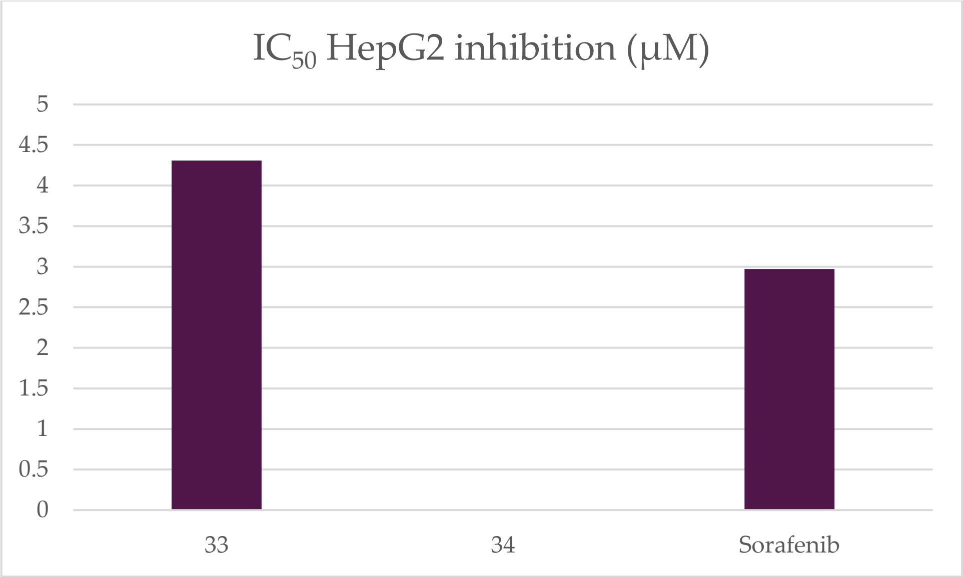

Figure S15. IC_{50} values for HepG2 inhibition by compounds 33 and 34 reported by El-Hazek, R.M.M et al. [48]

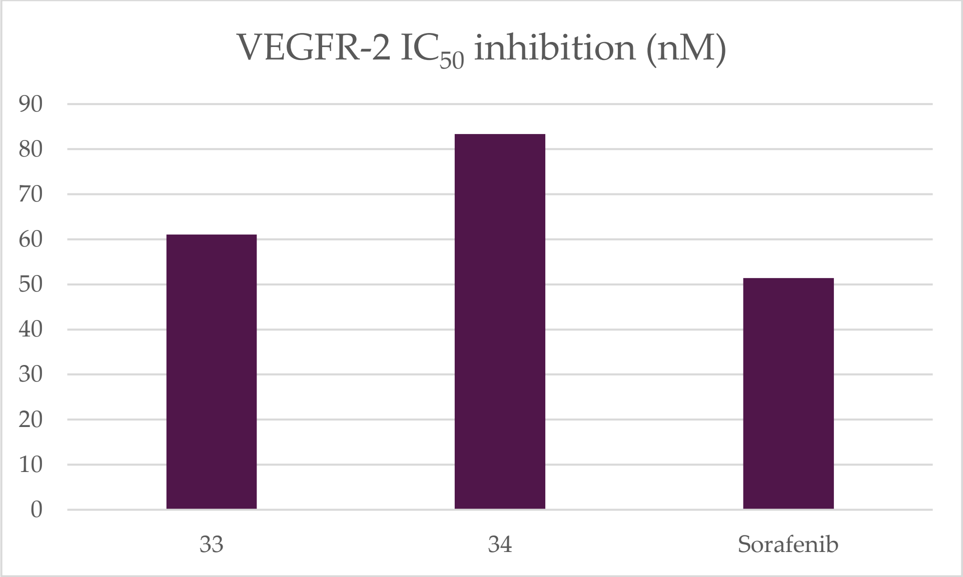

Figure S16. VEGFR-2 IC_{50} inhibition for compounds 33 and 34 reported by El-Hazek, R.M.M et al. [48]

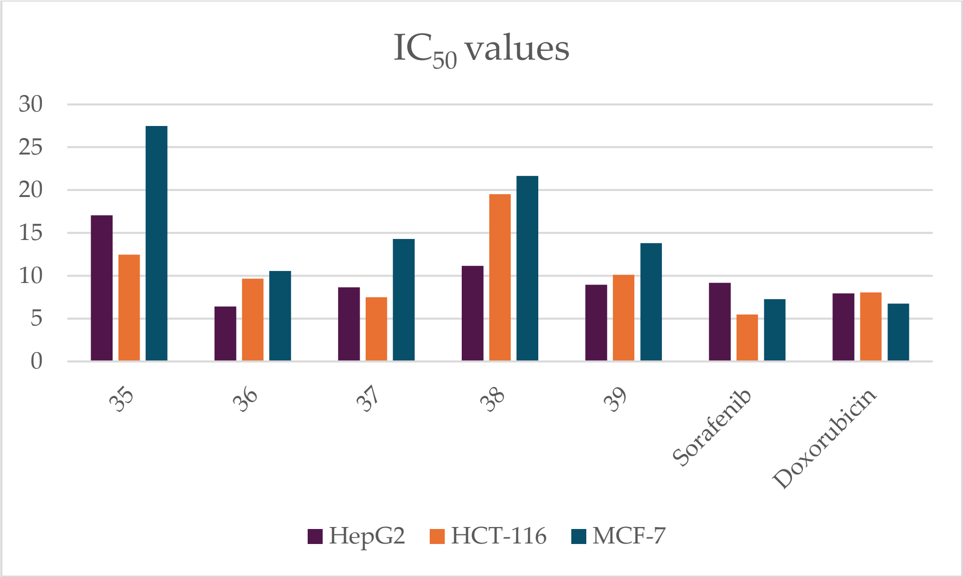

Figure S17. IC_{50} values for HepG2, HCT-116 and MCF-7 inhibition by compounds 35-39 reported by Sayed, A.M et al. [49]

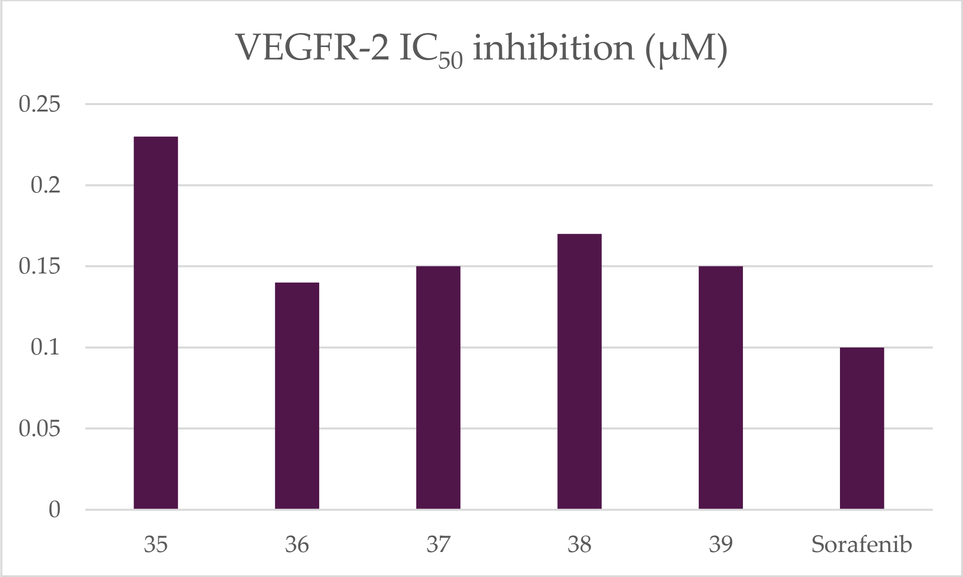

Figure S18. VEGFR-2 IC_{50} inhibition for compounds 35-39 reported by Sayed, A.M et al. [49]

Table S1. Consolidated table of sulfonamides, bioassays performed and results on the most promising compounds and used reference compounds.

Compound	Synthesized by	Cytotoxic assay	IC_{50}	Enzyme Inhibition Assay	K_I	Receptor Inhibition Assay	IC_{50}
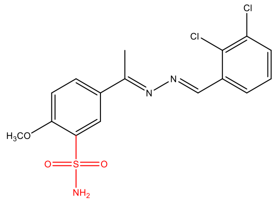	**1** [42]	MTT assay against human tumor cell lines; HepG2 and MCF-7	0.15 μM 0.09 μM, respectively	CA IX and CA XII	K_I > 100 μM	VEGFR-2	23.1 ± 0.75 nM
		Reference: Staurosporine	10.42 μM (HepG2) 3.10 μM (MCF-7)	Reference: Acetazolamide	0.025 μM (CA IX) 0.006 μM (CA XII)	Reference: Sorafenib	29.7 ± 0.17 nM
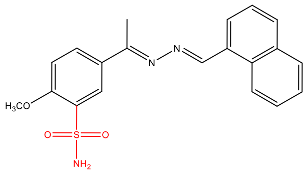	**2** [42]	MTT assay against human tumor cell lines; HepG2 and MCF-7	0.15 μM 0.26 μM, respectively	CA IX and CA XII	K_I > 100 μM	VEGFR-2	31.1 ± 0.75 nM
		Reference: Staurosporine	10.42 μM (HepG2) 3.10 μM (MCF-7)	Reference: Acetazolamide	0.025 μM (CA IX) 0.006 μM (CA XII)	Reference: Sorafenib	29.7 ± 0.17 nM
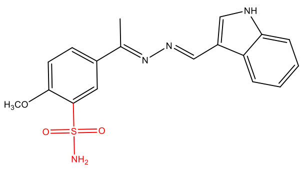	**3** [42]	MTT assay against human tumor cell lines; HepG2 and MCF-7	1.55 μM 1.11 μM, respectively data	CA IX and CA XII	K_I > 100 μM	VEGFR-2	40.1 ± 0.90 nM
		Reference: Staurosporine	10.42 μM (HepG2) 3.10 μM (MCF-7)	Reference: Acetazolamide	0.025 μM (CA IX) 0.006 μM (CA XII)	Reference: Sorafenib	29.7 ± 0.17 nM
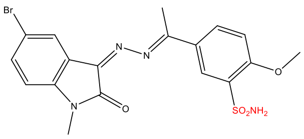	**4** [43]	Sulforhodamine B colorimetric assay against T47D breast cancer cells	10.40 ± 0.47 μM	CA I, CA II and CA IX	K_I > 100 μM	VEGFR-2	30.10 ± 0.31 nM
		Reference: Doxorubicin	2.26 ± 0.10 μM	Reference: Acetazolamide	0.25 (CA I) 0.012 (CA II) 0.026 (CA IX)	Reference: Sorafenib	29.70 ± 0.17 nM

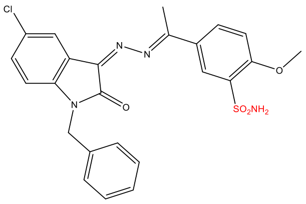	**5** [43]	Sulforhodamine B colorimetric assay against T47D breast cancer cells	3.59 ± 0.16 µM	CA I, CA II and CA IX	K_I > 100 µM	VEGFR-2	23.10 ± 0.41 nM
		Reference: Doxorubicin	2.26 ± 0.10 µM	Reference: Acetazola mide	0.25 (CA I) 0.012 (CA II) 0.026 (CA IX)	Reference: Sorafenib	29.70 ± 0.17 nM
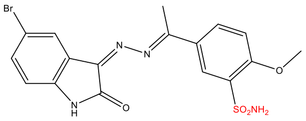	**7** [43]	Sulforhodamine B colorimetric assay against T47D breast cancer cells	5.45 ± 0.24 µM	CA I, CA II and CA IX	K_I > 100 µM	VEGFR-2	56.70 ± 0.72 nM
		Reference: Doxorubicin	2.26 ± 0.10 µM	Reference: Acetazola mide	0.25 (CA I) 0.012 (CA II) 0.026 (CA IX)	Reference: Sorafenib	29.70 ± 0.17 nM
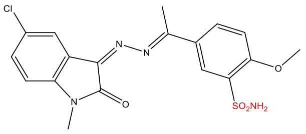	**9** [43]	Sulforhodamine B colorimetric assay against T47D breast cancer cells	1.83 ± 0.08 µM	CA I, CA II and CA IX	K_I > 100 µM	VEGFR-2	63.40 ± 0.72 nM
		Reference: Doxorubicin	2.26 ± 0.10 µM	Reference: Acetazola mide	0.25 (CA I) 0.012 (CA II) 0.026 (CA IX)	Reference: Sorafenib	29.70 ± 0.17 nM
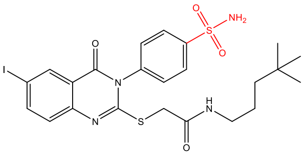	**12** [44]	MTT assay against human tumor cell lines; HepG2, MCF-7, HCT-116, and A549	0.1163 ± 0.02, 0.4092 ± 0.02, 0.1985 ± 0.02 and 1.8986 ± 0.25 µM respectively			VEGFR-2 and EGFR^{T790M}	0.2193 ± 0.02 µM and NT respectively
		Reference: Sorafenib and Erlotinib	0.400 ± 0.03 (HepG2) and 0.773 ± 0.07 µM (HepG2) respectively 0.404 ± 0.03 (MCF-7) and 0.549 ± 0.05 µM (MCF-7) respectively 0.558 ± 0.05 (HCT-116) and			Reference: Sorafenib (VEGFR-2) and Erlotinib (EGFR^{T790M})	0.1400 ± 0.01 and 0.2420 ± 0.02 µM respectivel y

			0.820 ± 0.06 µM (HCT-116) respectively<br>0.505 ± 0.05 (A549) and 0.1391 ± 0.01 (A549) respectively			
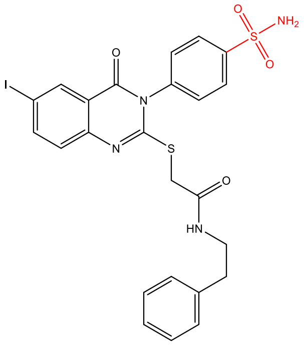	**14** [44]	MTT assay against human tumor cell lines; HepG2, MCF-7, HCT-116, and A549<br>Reference: Sorafenib and Erlotinib	0.1707 ± 0.02, 0.4620 ± 0.02, 0.3792 ± 0.02 and 0.4511 ± 0.05 µM respectively<br>0.400 ± 0.03 (HepG2) and 0.773 ± 0.07 µM (HepG2) respectively<br>0.404 ± 0.03 (MCF-7) and 0.549 ± 0.05 µM (MCF-7) respectively<br>0.558 ± 0.05 (HCT-116) and 0.820 ± 0.06 µM (HCT-116) respectively<br>0.505 ± 0.05 (A549) and 0.1391 ± 0.01 µM (A549) respectively		VEGFR-2 and EGFR^{T790M}<br>Reference: Sorafenib (VEGFR-2) and Erlotinib (EGFR^{T790M})	0.2510 ± 0.02 and 0.3898 ± 0.02 µM respectively<br>0.1400 ± 0.01 and 0.2420 ± 0.02 µM respectively

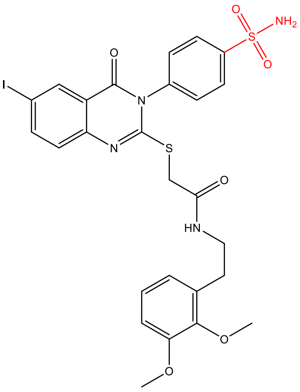	**15** [44]	MTT assay against human tumor cell lines; HepG2, MCF-7, HCT-116, and A549	0.2232 ± 0.02, 0.6939 ± 0.07, 0.5872 ± 0.05 and 0.7887 ± 0.07 µM respectively	VEGFR-2 and $EGFR^{T790M}$	0.3931 ± 0.02 and 0.6615 ± 0.05 µM respectively
		Reference: Sorafenib and Erlotinib	0.400 ± 0.03 (HepG2) and 0.773 ± 0.07 µM (HepG2) respectively 0.404 ± 0.03 (MCF-7) and 0.549 ± 0.05 µM (MCF-7) respectively 0.558 ± 0.05 (HCT-116) and 0.820 ± 0.06 µM (HCT-116) respectively 0.505 ± 0.05 (A549) and 0.1391 ± 0.01 µM (A549) respectively	Reference: Sorafenib (VEGFR-2) and Erlotinib ($EGFR^{T790M}$)	0.1400 ± 0.01 and 0.2420 ± 0.02 µM respectively
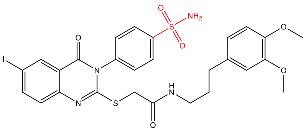	**17** [44]	MTT assay against human tumor cell lines; HepG2, MCF-7, HCT-116, and A549	1.1923 ± 0.10, 0.1781 ± 0.02, 0.1451 ± 0.01 and 1.7428 ± 0.15 µM respectively	VEGFR-2 and $EGFR^{T790M}$	0.0984 ± 0.01 µM and NT respectively
		Reference: Sorafenib and Erlotinib	0.400 ± 0.03 (HepG2) and 0.773 ± 0.07 µM (HepG2) respectively	Reference: Sorafenib (VEGFR-2) and Erlotinib	0.1400 ± 0.01 and 0.2420 ± 0.02 µM

			0.404 ± 0.03 (MCF-7) and 0.549 ± 0.05 μM (MCF-7) respectively 0.558 ± 0.05 (HCT-116) and 0.820 ± 0.06 μM (HCT-116) respectively 0.505 ± 0.05 (A549) and 0.1391 ± 0.01 (A549) respectively	(EGFR^{T790M})	respectively
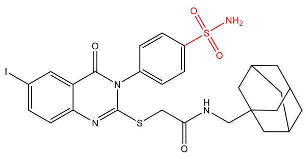	**22** [44]	MTT assay against human tumor cell lines; HepG2, MCF-7, HCT-116, and A549<br>Reference: Sorafenib and Erlotinib	0.3076 ± 0.02, 0.4029 ± 0.05, 1.1217 ± 0.15 and 0.8211 ± 0.07 μM respectively<br>0.400 ± 0.03 (HepG2) and 0.773 ± 0.07 μM (HepG2) respectively 0.404 ± 0.03 (MCF-7) and 0.549 ± 0.05 μM (MCF-7) respectively 0.558 ± 0.05 (HCT-116) and 0.820 ± 0.06 μM (HCT-116) respectively 0.505 ± 0.05 (A549) and 0.1391 ± 0.01 μM (A549) respectively	VEGFR-2 and EGFR^{T790M}<br>Reference: Sorafenib (VEGFR-2) and Erlotinib (EGFR^{T790M})	0.2174 ± 0.02 and 0.3516 ± 0.02 μM respectively<br>0.1400 ± 0.01 and 0.2420 ± 0.02 μM respectively
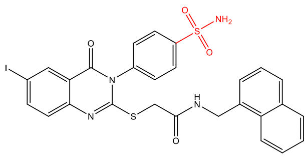	**23** [44]	MTT assay against human tumor cell lines; HepG2, MCF-7, HCT-116, and A549	0.3425 ± 0.02, 0.0977 ± 0.01, 0.2000 ± 0.02 and 0.5134 ± 0.05 μM respectively	VEGFR-2 and EGFR^{T790M}	0.0523 ± 0.01 and 0.0728 ± 0.01 μM respectively

		Reference: Sorafenib and Erlotinib	0.400 ± 0.03 (HepG2) and 0.773 ± 0.07 µM (HepG2) respectively 0.404 ± 0.03 (MCF-7) and 0.549 ± 0.05 µM (MCF-7) respectively 0.558 ± 0.05 (HCT-116) and 0.820 ± 0.06 µM (HCT-116) respectively 0.505 ± 0.05 (A549) and 0.1391 ± 0.01 µM (A549) respectively			Reference: Sorafenib (VEGFR-2) and Erlotinib ($EGFR^{T790M}$)	0.1400 ± 0.01 and 0.2420 ± 0.02 µM respectively
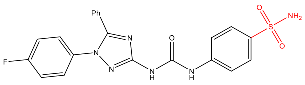	**25** [45]	MTT assay against human tumor cell lines; MCF-7 and T47D	0.66 ± 0.04 and 4.51 ± 0.2 µM respectively	*h*CA IX and *h*CA XII	K_I: 8.3 and 4.7 nM respectively	VEGFR-2	26.3 ± 0.4 nM
		Reference: Staurosporine	3.18 ± 0.18 (MCF-7) and 7.12 ± 0.31 µM (T47D)	Reference: Acetazolamide	25.0 (*h*CA IX) and 5.7 nM (*h*CA XII)	Reference: Sunitinib	39.7 ± 2 nM
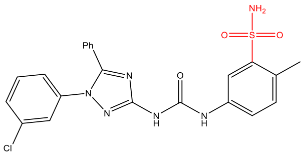	**26** [45]	MTT assay against human tumor cell lines; MCF-7 and T47D	10.9 ± 0.62 and 17.9 ± 0.78 µM respectively	*h*CA IX and *h*CA XII	K_I: 67.2 and 61.0 nM respectively	VEGFR-2	96.2 ± 2 nM
		Reference: Staurosporine	3.18 ± 0.18 (MCF-7) and 7.12 ± 0.31 µM (T47D)	Reference: Acetazolamide	25.0 (*h*CA IX) and 5.7 nM (*h*CA XII)	Reference: Sunitinib	39.7 ± 2 nM
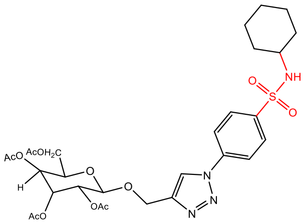	**30** [46]	MTT assay against human tumor cell lines; A-549, HepG2, MCF-7 and HCT-116	20.45±0.28, 10.45±0.13, 20.31±0.66 and 32.05±0.42 µM respectively	*h*CA IX and *h*CA XII	IC_{50}: 66 and 7.6 nM respectively	VEGFR-2	1.33±0.10 µM

		Reference: Doxorubicin (HepG2, MCF-7) and Sunitinib (A-549, HCT-116)	13.76±0.45 (HepG2), 17.44±0.46 (MCF-7), 10.14±0.50 (A-549) and 9.67±0.22 µM (HCT-116)	Reference: SLC-0111	53 (*h*CA IX) 4.8 nM (*h*CA XII)	Reference: Sorafenib	0.43±0.10 µM
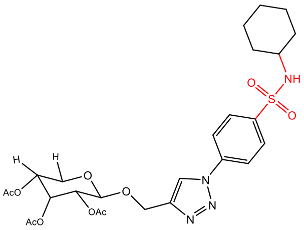	**31** [46]	MTT assay against human tumor cell lines; A-549, HepG2, MCF-7 and HCT-116	19.81±0.65, 8.39±0.20, 21.15±2.45 and 23.60±0.22 µM respectively	*h*CA IX and *h*CA XII	IC_{50}: 40 and 3.2 nM respectively	VEGFR-2	0.38±0.14 µM
		Reference: Doxorubicin (HepG2, MCF-7) and Sunitinib (A-549, HCT-116)	13.76±0.45 (HepG2), 17.44±0.46 (MCF-7), 10.14±0.50 (A-549) and 9.67±0.22 µM (HCT-116)	Reference: SLC-0111	53 (*h*CA IX) 4.8 nM (*h*CA XII)	Reference: Sorafenib	0.43±0.10 µM
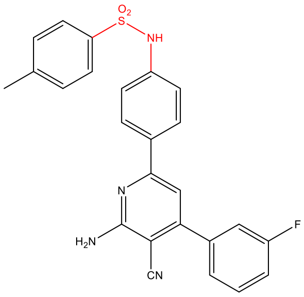	**32** [47]	NCI panel five dose assay, against 60 lines of human cancer cells	GI50 values between 1.06 and 8.92 mM			VEGFR-2	3.62 ± 0.04 µM
						Reference: Sorafenib	4.58 ± 0.05 µM
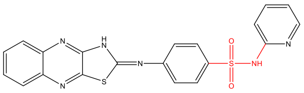	**33** [48]	Cytotoxic anticancer activity against human HepG2 cell line	4.31 µM			VEGFR-2	61.04±2.60 nM
		Reference: Sorafenib	2.97 µM			Reference: Sorafenib	51.41±2.30 nM
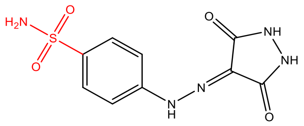		MTT assay against human tumor cell lines; HepG2, HCT-116 and MCF-7	17.06 ± 1.5, 12.48 ± 1.1 and 27.48 ± 2.2 µM respectively			VEGFR-2	0.23 ± 0.03 µM

	**35** [49]	Reference: Sorafenib and Doxorubicin	9.18 ± 0.6 (HepG2) and 7.94 ± 0.6 µM (HepG2) respectively 5.47 ± 0.3 (HCT-116) and 8.07 ± 0.8 µM (HCT-116) respectively 7.26 ± 0.3 (MCF-7) and 6.75 ± 0.4 µM (MCF-7) respectively	Reference: Sorafenib	0.10 ± 0.02 µM
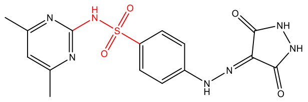	**36** [49]	MTT assay against human tumor cell lines; HepG2, HCT-116 and MCF-7	6.43 ± 0.5, 9.66 ± 0.8 and 10.57 ± 0.9 µM respectively	VEGFR-2	0.14 ± 0.02 µM
		Reference: Sorafenib and Doxorubicin	9.18 ± 0.6 (HepG2) and 7.94 ± 0.6 µM (HepG2) respectively 5.47 ± 0.3 (HCT-116) and 8.07 ± 0.8 µM (HCT-116) respectively 7.26 ± 0.3 (MCF-7) and 6.75 ± 0.4 µM (MCF-7) respectively	Reference: Sorafenib	0.10 ± 0.02 µM
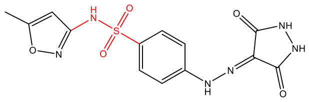	**37** [49]	MTT assay against human tumor cell lines; HepG2, HCT-116 and MCF-7	8.65 ± 0.7, 7.49 ± 0.6 and 14.29 ± 1.3 µM respectively	VEGFR-2	0.15 ± 0.02 µM
		Reference: Sorafenib and Doxorubicin	9.18 ± 0.6 (HepG2) and 7.94 ± 0.6 µM (HepG2) respectively 5.47 ± 0.3 (HCT-116) and 8.07 ±	Reference: Sorafenib	0.10 ± 0.02 µM

			0.8 µM (HCT-116) respectively 7.26 ± 0.3 (MCF-7) and 6.75 ± 0.4 µM (MCF-7) respectively		
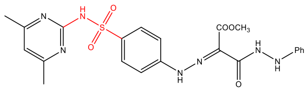	**38** [49]	MTT assay against human tumor cell lines; HepG2, HCT-116 and MCF-7	11.17 ± 1.0, 19.52 ± 1.7 and 21.65 ± 1.9 µM respectively	VEGFR-2	0.17 ± 0.02 µM
		Reference: Sorafenib and Doxorubicin	9.18 ± 0.6 (HepG2) and 7.94 ± 0.6 µM (HepG2) respectively 5.47 ± 0.3 (HCT-116) and 8.07 ± 0.8 µM (HCT-116) respectively 7.26 ± 0.3 (MCF-7) and 6.75 ± 0.4 µM (MCF-7) respectively	Reference: Sorafenib	0.10 ± 0.02 µM
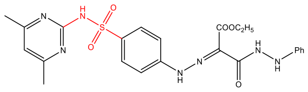	**39** [49]	MTT assay against human tumor cell lines; HepG2, HCT-116 and MCF-7	8.97 ± 0.7, 10.13 ± 0.9 and 13.82 ± 1.1 µM respectively	VEGRF-2	0.15 ± 0.02 µM
		Reference: Sorafenib and Doxorubicin	9.18 ± 0.6 (HepG2) and 7.94 ± 0.6 µM (HepG2) respectively 5.47 ± 0.3 (HCT-116) and 8.07 ± 0.8 µM (HCT-116) respectively 7.26 ± 0.3 (MCF-7) and 6.75 ± 0.4 µM (MCF-7) respectively	Reference: Sorafenib	0.10 ± 0.02 µM

NT: not tested compounds
